# Weight gain leads to greater adverse metabolic responses in South Asian compared with white European men: the GlasVEGAS study

**DOI:** 10.1038/s42255-024-01101-z

**Published:** 2024-08-16

**Authors:** James McLaren, Xuan Gao, Nazim Ghouri, Dilys J. Freeman, Janice Richardson, Naveed Sattar, Jason M. R. Gill

**Affiliations:** 1 School of Cardiovascular and Metabolic Health, College of Medical Veterinary and Life Sciences, University of Glasgow, Glasgow, UK; 2 Department of General Medicine, Queen Elizabeth University Hospital, Glasgow, UK

**Keywords:** Obesity, Diabetes, Metabolic diseases, Translational research

## Abstract

South Asians (SAs) develop type 2 diabetes at lower body mass index values than white Europeans (WEs). This basic human experimental study aimed to compare the metabolic consequences of weight gain in SA and WE men without overweight or obesity. Fourteen SAs and 21 WEs had assessments of body composition, metabolic responses to mixed-meal ingestion, cardiorespiratory fitness and physical activity, and a subcutaneous abdominal adipose tissue biopsy, before and after 4–6 weeks of overfeeding to induce 5–7% weight gain. Here we show that body mass index and whole-body adipose tissue volume increases similarly between ethnic groups, but SAs gain less lean tissue. SAs experience a substantially greater decrease in insulin sensitivity compared with WEs (38% versus 7% decrease, *P* = 0.009), have fewer small (37.1% versus 60.0%, *P* = 0.003) and more large (26.2% versus 9.1%, *P* = 0.005) adipocytes at baseline and have a smaller decrease in very small adipocytes with weight gain (−0.1% versus −1.9%, *P* < 0.0001). Ethnic differences in adipocyte morphology are associated with SA’s greater adverse metabolic changes with weight gain. ClinicalTrials.gov registration: NCT02399423.

## Abstract (web summary)

Modest weight gain leads to greater adverse metabolic consequences in South Asian compared to European men, in part driven by differences in adipocyte morphology.

## Main

SAs have three- to fivefold higher type 2 diabetes prevalence, and develop the disease a decade earlier and at lower body mass index (BMI) values, than WEs^1^. The increment in diabetes risk per unit difference in BMI is substantially greater in SAs than WEs^2,3^, suggesting greater adverse metabolic consequences of increasing adiposity. For a given BMI, SAs typically have higher percentage body fat and lower muscle mass than WEs^4^. They also have larger subcutaneous abdominal adipocytes—which is associated with their greater insulin resistance^5,6^—as well as higher liver fat, at equivalent BMI^7^. Thus, differences in adipocyte morphology and/or capacity for fat storage in ‘safe’ subcutaneous depots, leading to earlier ectopic fat accumulation, may underlie SA’s higher metabolic risk^1^.

However, existing data are largely cross-sectional, and it is unclear whether differences exist in the metabolic consequences of weight gain between individuals of SA versus WE descent. One study reported that short-term (5 days) overfeeding with a high-fat diet reduced insulin sensitivity in young, lean SAs but not in matched WEs^8^. However, no significant changes in subcutaneous or visceral fat were observed, and hepatic triglyceride content increased similarly in both groups, suggesting that this short-term effect was likely to be due to the acute effects of positive energy balance or high fat intake, rather than effects on adiposity. In contrast, another small study reported similar changes to the metabolic profile in response to 4 days of overfeeding with a high-fat diet in young SA and WE men, again with similar changes to liver fat accumulation in the two ethnic groups^9^. While these data suggest that SAs may have increased susceptibility to the adverse effects of a ‘Westernized’ lifestyle, the effects of short-term positive energy balance are not necessarily analogous to weight gain, and thus the longer-term effects of established weight gain are unclear. The aims of this basic human experimental study were, therefore, to compare the effects of induced weight gain in SA and WE men without overweight or obesity at baseline on: (i) body composition and subcutaneous, visceral and ectopic fat accumulation; (ii) metabolic responses to a physiologically relevant mixed-meal challenge; and (iii) adipocyte morphology. We also sought to investigate factors contributing to ethnic differences in weight-gain-induced changes in indices of insulin sensitivity/insulin resistance and estimated insulin secretion/beta cell function, and we undertook an exploratory analysis of ethnic differences in expression of adipocyte genes related to lipid metabolism, differentiation, tissue stress response and inflammation.

## Results

**Fig. 1:**
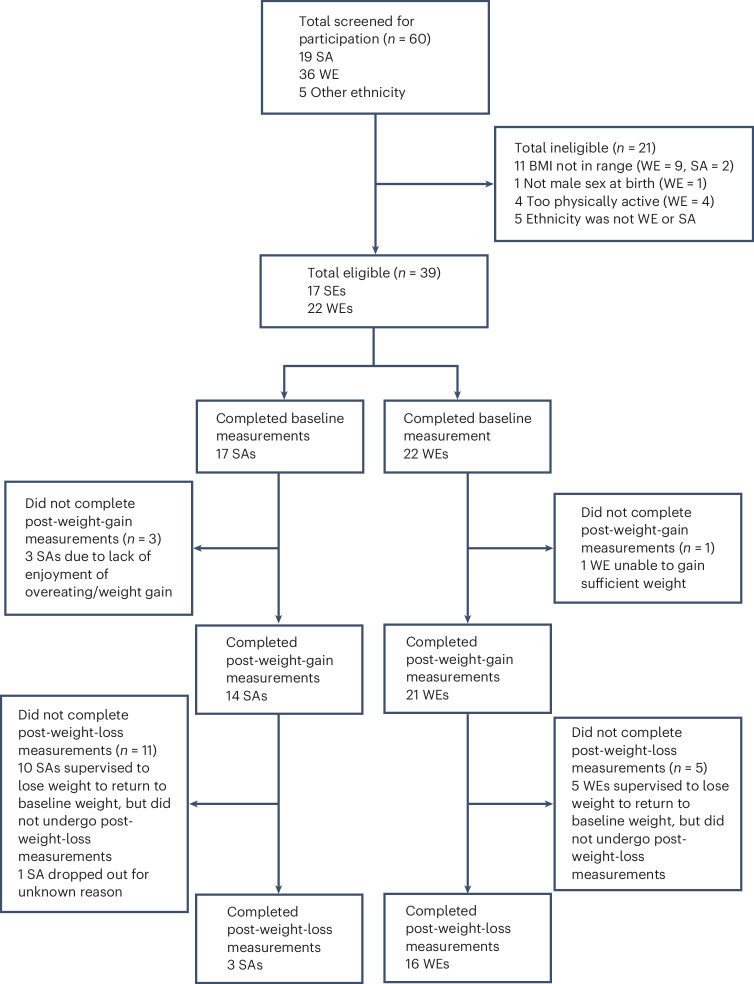
GlasVEGAS study CONSORT diagram.

Figure 1 shows participant flow throughout the study. Seventeen SA and 22 WE participants were recruited and completed baseline measurements, and 14 SA men and 21 WE men completed weight gain assessments. Three SA and one WE participant reported having a first-degree relative with type 2 diabetes. Data are presented for participants who completed both baseline and post-weight-gain measurements. For variables where there were missing data, the number of participants where data were available is reported separately in tables and figures. Only 3 SA and 16 WE participants completed measurements following the weight loss phase of the study; therefore, these data are not robust and have not been presented.

### Anthropometric and body composition data

Table 1 shows age, anthropometric and body composition data at baseline (that is, before intervention) and in response to weight gain in the two groups. There were no differences in age or BMI between groups at baseline (by design). Increases in weight and BMI with the weight gain intervention were similar between groups, with the WE men gaining 6.3% ± 0.2% and SA men gaining 6.5% ± 0.3% in body weight in response to the intervention (*P* = 0.62 for ethnic difference).

**Table 1:** Age and anthropometric/body composition in WE and SA men at baseline and after weight gain

	Baseline			Change with weight gain				
	WE (*n* = 21)	SA (*n* = 14)	*P*_baseline ethnicity_	WE (*n* = 21)	SA (*n* = 14)	*P*_weight gain_	*P*_ethnicity × __weight gain_	*P*_ethnicity × weight gain (adjusted for baseline)*_
**Age and anthropometric/body composition data**								
Age (years)	22.1 ± 0.7	23.1 ± 0.9	0.35	-	-	-	-	-
Height (m)	1.83 ± 0.01	1.77 ± 0.02	0.02	-	-	-	-	-
Weight (kg)	74.7 ± 1.7	67.8 ± 1.9	0.01	4.7 ± 0.2	4.4 ± 0.2	<0.0001	0.40	0.62
BMI (kg m^−2^)	22.3 ± 0.3	21.7 ± 0.7	0.45	1.41 ± 0.06	1.41 ± 0.08	<0.0001	0.97	0.62
Waist (cm)	78.2 ± 0.9	76.7 ± 1.3	0.33	3.8 ± 0.4	5.2 ± 0.8	<0.0001	0.12	0.15
Whole-body lean tissue (l)^a^	44.0 ± 1.2	39.2 ± 1.2	0.01	2.4 ± 0.5	0.7 ± 0.3	<0.0001	0.02	0.004
Whole-body adipose tissue (l)^a^	14.2 ± 0.8	18.3 ± 1.1	0.005	3.1 ± 0.3	3.0 ± 0.3	<0.0001	0.72	0.41
Upper-body adipose tissue (l)^a^	6.3 ± 0.5	8.3 ± 0.7	0.008	1.8 ± 0.2	1.7 ± 0.2	<0.0001	0.64	0.40
Lower-body adipose tissue (l)^a^	7.9 ± 0.4	9.7 ± 0.5	0.009	1.3 ± 0.2	1.3 ± 0.2	<0.0001	0.85	0.44
ASAT (l)^a^	2.5 ± 0.2	4.0 ± 0.4	0.0007	0.8 ± 0.1	1.0 ± 0.1	<0.0001	0.28	0.98
VAT (l)	1.1 ± 0.1	1.4 ± 0.2	0.27	0.6 ± 0.1	0.3 ± 0.1	<0.0001	0.004	0.002
Liver fat fraction (%)^b^	2.1 ± 0.3	4.1 ± 1.0	0.03	1.0 ± 0.9	3.6 ± 3.3	0.10	0.36	0.74
Muscle fat fraction (%)^c^	2.4 ± 0.2	3.1 ± 0.4	0.12	0.6 ± 0.4	0.0 ± 0.5	0.33	0.38	0.84
VAT/ASAT ratio^a^	0.45 ± 0.03	0.34 ± 0.04	0.03	0.06 ± 0.01	0.00 ± 0.01	0.001	<0.0001	<0.0001

Values are mean ± s.e.m.

*P* for statistical significance adjusted from 0.05 to 0.02 to control the familywise error rate for multiple comparisons using the method described by Tukey et al.^45^.

*P*_baseline ethnicity_ difference between WEs and SAs at baseline (unpaired *t*-test, two-sided).

*P*_weight gain_ main effect of weight gain intervention (two-way analysis of variance (ANOVA), two-sided).

*P*_ethnicity × __weight gain_ ethnicity × weight gain intervention interaction (two-way ANOVA, two-sided).

*P_baseline value × __weight gain_ ethnicity × weight gain intervention interaction (two-way analysis of covariance (ANCOVA), adjusted for baseline values, two-sided).

^a^*n* = 20 WEs, 13 SAs; ^b^*n* = 20 WEs, 12 SAs; ^c^*n* = 15 WEs, 10 SAs.

At baseline, SA men had less whole-body lean tissue, and more whole-body, upper-body, lower-body and abdominal subcutaneous adipose tissue (ASAT) than WE men. Ectopic fat in the liver, but not muscle, was higher in SA men. Visceral adipose tissue (VAT) volume did not differ significantly between ethnic groups, and the VAT/ASAT ratio was higher in WE men.

There were similar, statistically significant, increases in whole-body, upper-body and lower-body adipose tissue and ASAT with weight gain in both ethnic groups. However, the increases in VAT, the VAT/ASAT ratio and whole-body lean tissue were greater in WE men. There was considerable between-individual variability in the change in liver fat and muscle fat fractions in response to the intervention and, overall, neither changed significantly with weight gain.

### Metabolic data

**Fig. 2:**
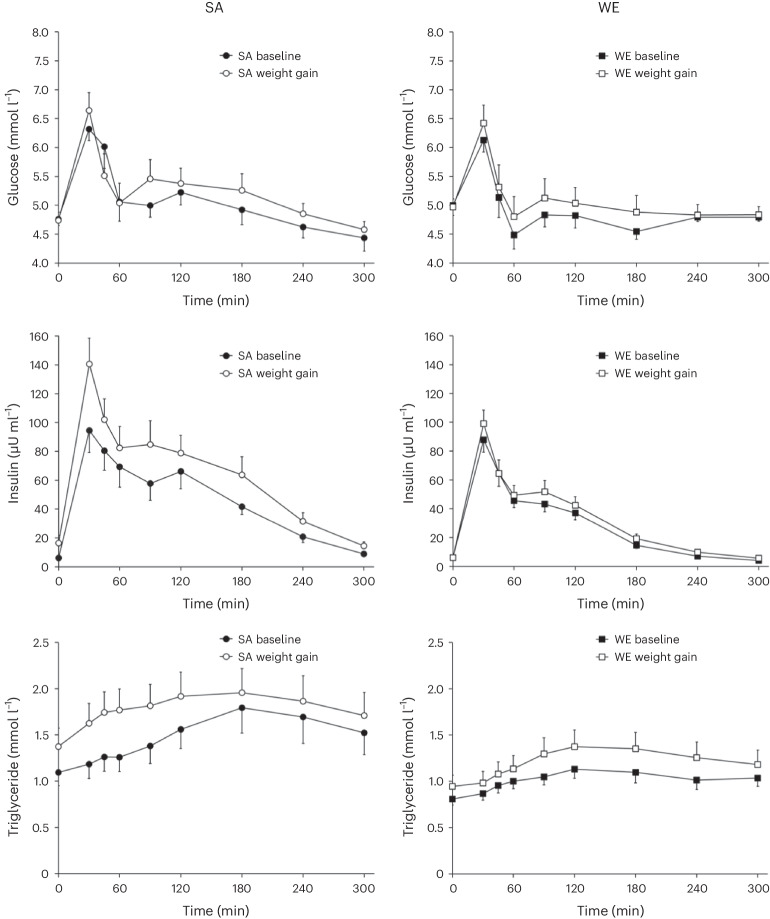
Postprandial glucose, insulin and triglyceride responses to a mixed meal in SA (*n* = 14) and WE (*n* = 21) men at baseline and following the weight gain intervention. Summary measures of these responses are reported in Table 2. Values are the mean ± s.e.m. Source data

Summarized fasting and postprandial metabolic data at baseline and in response to weight gain are shown in Table 2, with Fig. 2 presenting metabolic responses over the 300-min postprandial observation period.

**Table 2:** Metabolic, physical activity and fitness data in WE and SA men at baseline and after weight gain

	Baseline			Change with weight gain				
	WE (*n* = 21)	SA (*n* = 14)	*P*_baseline ethnicity_	WE (*n* = 21)	SA (*n* = 14)	*P*_weight gain_	*P*_ethnicity × __weight gain_	*P*_ethnicity × __weight gain (adjusted for baseline)*_
**Metabolic data**								
Fasting glucose (mmol l^−1^)	5.00 ± 0.17	4.76 ± 0.11	0.30	−0.03 ± 0.20	−0.02 ± 0.12	0.86	0.97	0.31
Postprandial glucose (mmol l^−1^)	4.86 ± 0.12	5.02 ± 0.16	0.38	0.19 ± 0.13	0.19 ± 0.22	0.11	1.00	0.67
Fasting insulin (µU ml^−1^)	6.1 ± 1.1	6.0 ± 0.9	0.38	0.0 ± 1.0	10.5 ± 5.1	0.02	0.02	0.02
Postprandial insulin (µU ml^−1^)	28.2 ± 2.7	45.6 ± 6.1	0.006	4.4 ± 2.5	17.8 ± 5.0	<0.0001	0.01	0.09
HOMA_IR_	1.32 ± 0.22	1.34 ± 0.22	0.95	0.05 ± 0.21	2.15 ± 0.95	0.01	0.01	0.01
Matsuda insulin sensitivity index	8.00 ± 0.68	7.34 ± 1.16	0.61	−0.58 ± 0.65	−2.77 ± 0.47	0.001	0.02	0.009
Insulin AUC/glucose AUC	5.79 ± 0.52	9.08 ± 1.21	0.008	0.59 ± 0.43	2.76 ± 0.89	0.001	0.02	0.03
ISSI-2	40.8 ± 2.1	55.8 ± 5.1	0.004	−2.0 ± 2.4	−15.1 ± 5.1	0.002	0.01	0.78
Fasting triglycerides (mmol l^−1^)	0.81 ± 0.07	1.09 ± 0.14	0.05	0.14 ± 0.07	0.28 ± 0.13	0.006	0.32	0.57
Postprandial triglycerides (mmol l^−1^)	1.03 ± 0.09	1.52 ± 0.22	0.02	0.20 ± 0.09	0.29 ± 0.15	0.005	0.58	0.77
Resting metabolic rate (kcal day^−1^)	1,644 ± 57	1,501 ± 67	0.11	111 ± 33	168 ± 58	<0.0001	0.37	0.84
Resting metabolic rate (kcal kg^−1^day^−1^)	21.9 ± 0.5	22.1 ± 0.8	0.82	0.1 ± 0.4	1.1 ± 0.8	0.15	0.26	0.20
**Cardiorespiratory fitness and physical activity data**								
Maximal oxygen uptake (l min^−1^)^a^	3.84 ± 0.13	2.92 ± 0.11	<0.0001	−0.07 ± 0.06	0.00 ± 0.06	0.54	0.43	0.22
Maximal oxygen uptake (ml kg^−1^ min^−1^)^a^	51.2 ± 1.2	44.0 ± 0.9	0.0001	−3.2 ± 0.7	−2.4 ± 0.9	<0.0001	0.49	0.73
Accelerometer wear time (min day^−1^)^b^	812 ± 28	720 ± 43	0.08	−75 ± 27	6.6 ± 66	0.27	0.19	0.49
Time sedentary (min day^−1^)^b^	610 ± 31	529 ± 35	0.14	−45 ± 26	52 ± 56	0.90	0.09	0.22
Time in light activity (min day^−1^)^b^	139 ± 15	131 ± 13	0.72	−25 ± 11	−35 ± 11	0.015	0.60	0.35
Time in moderate activity (min day^−1^)^b^	54 ± 7	49 ± 7	0.69	−5 ± 9	−6 ± 5	0.42	0.93	0.59
Time in vigorous activity (min day^−1^)^b^	9 ± 3	11 ± 6	0.71	0 ± 1	−4 ± 5	0.31	0.26	0.27
Steps per day^b^	8,631 ± 828	8,714 ± 873	0.95	−892 ± 996	−2,050 ± 640	0.08	0.47	0.39

Values are the mean ± s.e.m.

*P* for statistical significance adjusted from 0.05 to 0.02 to control the familywise error rate for multiple comparisons using the method described by Tukey et al.^45^.

*P*_baseline ethnicity_ difference between WEs and SAs at baseline (unpaired *t*-test, two-sided).

*P*_weight gain_ main effect of weight gain intervention (two-way ANOVA, two-sided).

*P*_ethnicity × __weight gain_ ethnicity × weight gain intervention interaction (two-way ANOVA, two-sided).

*P_baseline value × __weight gain_ ethnicity × weight gain intervention interaction (two-way ANCOVA, adjusted for baseline values, two-sided).

^a^*n* = 20 WEs, 12 SAs; ^b^*n* = 16 WEs, 7 SAs.

At baseline, there were no significant differences in fasting glucose, insulin or triglyceride between groups. However, postprandial insulin concentrations were 62% higher and postprandial triglyceride concentrations were 48% higher in SA participants. There were no differences in postprandial glucose concentrations at baseline between groups. The homeostatic model assessment of insulin resistance (HOMA_IR_) and the Matsuda insulin sensitivity index did not differ significantly between ethnic groups at baseline. However, insulin secretion, estimated from the insulin area under the curve (AUC)/glucose AUC ratio, and beta cell function, estimated from the insulin secretion sensitivity index 2 (ISSI-2) were significantly higher at baseline in SA participants. There were no differences in resting metabolic rate expressed either in absolute terms or per kilogram of body weight.

There was no significant effect of weight gain on fasting or postprandial glucose concentrations, but fasting and postprandial insulin and triglyceride concentrations all increased significantly in response to the weight gain intervention in the overall group. Overall, the Matsuda insulin sensitivity index decreased and HOMA_IR_ increased in response to weight gain. Estimated insulin secretion (insulin AUC/glucose AUC ratio) increased overall, and ISSI-2 decreased. Resting metabolic rate in absolute terms (kcal day^−1^) increased with weight gain but did not change with weight gain when expressed relative to body weight. However, the changes with weight gain in fasting and postprandial insulin were substantially greater in SA participants. Fasting insulin increased by 175% in SAs, compared to no increase in the WEs (*P* = 0.02 for ethnicity × weight gain interaction). This ethnicity × weight gain interaction remained significant after adjusting for baseline values (*P* = 0.02). The increase in postprandial insulin concentration with weight gain was more than fourfold higher, in terms of absolute concentrations, in SAs (39% relative increase from baseline) than WEs (15% relative increase from baseline; *P* = 0.01 for ethnicity × weight gain interaction). However, the ethnicity × weight gain interaction for the change in the postprandial insulin concentration was no longer statistically significant when adjusted for baseline values (*P* = 0.09), indicating that the greater increase postprandial insulin concentrations with weight gain observed in SA participants could be explained, in part, by their higher concentrations at baseline. Insulin sensitivity, as assessed by the Matsuda insulin sensitivity index, decreased by 38% in SAs, but only by 7% in the WEs, in response to weight gain (*P* = 0.02 for ethnicity × weight gain interaction). Similarly, HOMA_IR_ increased by 160% in SAs compared with 4% in WEs (*P* = 0.01 for ethnicity × weight gain interaction). The ethnicity × weight gain interactions for changes in the Matsuda insulin sensitivity index and HOMA_IR_ remained statistically significant after adjustment for baseline values. The increase in insulin AUC/glucose AUC ratio with weight gain was greater in SA than WE participants (*P* = 0.02). After adjustment for baseline values, the ethnicity × weight gain interaction just lost statistical significance after correction for multiple testing (*P* = 0.03). The ISSI-2 decreased more in SA than WE participants in response to weight gain (*P* = 0.01), but the ethnicity × weight gain interaction lost statistical significance after adjustment for baseline values (*P* = 0.78).

### Cardiorespiratory fitness and physical activity data

Table 2 shows cardiorespiratory fitness and physical activity data at baseline and following weight gain. At baseline, maximal oxygen uptake was lower in SA than WE participants both in absolute terms (l min^−1^; 24% lower, *P* < 0.0001) and relative to body weight (ml kg^−1^ min^−1^; 14% lower, *P* = 0.0001). Overall, maximal oxygen uptake expressed relative to body weight decreased similarly in response to weight gain in both ethnic groups but did not change with weight gain in absolute terms. There were no differences in any aspect of physical activity or sedentary time between the two groups at baseline. Overall, time spent in light activity decreased in response to weight gain, with no difference in this change between groups. There were no significant changes in sedentary time, daily step count or time spent in moderate or vigorous physical activity in response to the intervention.

### Subcutaneous adipocyte size and gene expression data

**Fig. 3:**
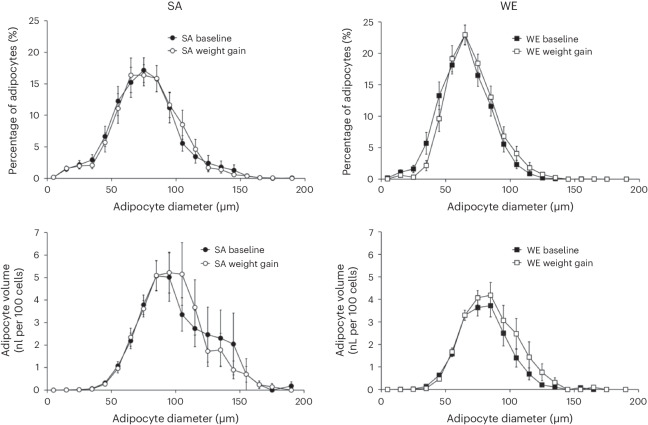
Distribution of proportion of adipocytes and adipocyte volume across the adipocyte size range in SA (*n* = 13) and WE (*n* = 17) men at baseline and following the weight gain intervention. Summary measures of these responses are reported in Table 3. Values are the mean ± s.e.m. Source data

Table 3 and Fig. 3 show subcutaneous abdominal adipocyte size and size distribution in the two ethnic groups at baseline and in response to weight gain. At baseline, mean adipocyte diameter (by 18%, *P* = 0.006) and adipocyte volume (by 76%, *P* = 0.006) were larger in SA participants. There was also an ethnic difference in the size distribution of adipocytes with SAs having fewer small adipocytes (*P* = 0.003) and more large adipocytes (*P* = 0.005) than WE participants. In SAs, 60% of their total adipocyte volume was contained in large adipocytes compared with 28% in WEs (*P* = 0.01). In contrast, only 12% of SA’s adipocyte volume was in small adipocytes, compared with 31% in WE participants (*P* = 0.002). There was considerable between-individual variability in changes in adipocyte size in response to the weight gain, which limited statistical power to detect changes in adipocyte size with weight gain. However, in analyses adjusted for baseline values, both the proportion and volume of very small adipocytes decreased more in response to weight gain in WE participants compared with SA participants (*P* < 0.0001 for both).

**Table 3:** Subcutaneous abdominal adipocyte size in WE and SA men at baseline and after weight gain

	Baseline			Change with weight gain				
	WE (*n* = 17)	SA (*n* = 13)	*P*_baseline ethnicity_	WE (*n* = 17)	SA (*n* = 13)	*P*_weight gain_	*P*_ethnicity × __weight gain_	*P*_ethnicity × w__eight gain (adjusted for baseline)*_
Mean adipocyte diameter (mm)	64.8 ± 2.2	76.3 ± 3.4	0.006	5.0 ± 2.1	1.4 ± 2.7	0.06	0.29	0.90
Adipocyte volume (nl per 100 cells)	17.98 ± 1.61	31.60 ± 4.87	0.006	3.89 ± 1.66	0.33 ± 3.80	0.28	0.36	0.77
Proportion of very small adipocytes (% cells)	2.9 ± 0.8	3.8 ± 1.0	0.48	−1.9 ± 0.8	−0.1 ± 1.1	0.16	0.20	<0.0001
Proportion of small adipocytes (% cells)	60.0 ± 4.1	37.1 ± 6.1	0.003	−6.2 ± 4.2	−1.9 ± 3.7	0.17	0.47	0.98
Proportion of medium adipocytes (% cells)	28.0 ± 3.1	33.0 ± 3.6	0.30	3.4 ± 3.1	−0.8 ± 3.8	0.59	0.40	0.85
Proportion of large adipocytes (% cells)	9.1 ± 2.2	26.2 ± 5.8	0.005	4.7 ± 2.3	2.8 ± 5.1	0.16	0.72	0.99
Volume of very small adipocytes (nl per 100 total cells)	0.02 ± 0.01	0.02 ± 0.01	0.52	−0.01 ± 0.01	0.00 ± 0.01	0.11	0.21	<0.0001
Volume of small adipocytes (nl per 100 total cells)	5.63 ± 0.25	3.64 ± 0.58	0.002	−0.16 ± 0.33	−0.01 ± 0.37	0.74	0.76	0.97
Volume of medium adipocytes (nl per 100 total cells)	7.36 ± 0.82	8.87 ± 0.98	0.25	0.90 ± 0.85	−0.16 ± 1.07	0.59	0.44	0.99
Volume of large adipocytes (nl per 100 total cells)	4.97 ± 1.23	19.06 ± 5.69	0.01	3.16 ± 1.50	0.50 ± 4.75	0.42	0.56	0.59

Values are the mean ± s.e.m.

Very small adipocytes, ≤30 μm diameter; small adipocytes, 31–70 μm; medium adipocytes, 71–90 μm; large adipocytes, >90 μm.

*P* for statistical significance adjusted from 0.05 to 0.02 to control the familywise error rate for multiple comparisons using the method described by Tukey et al.^45^.

*P*_baseline ethnicity_ difference between WEs and SAs at baseline (unpaired *t*-test, two-sided)

*P*_weight gain_ main effect of weight gain intervention (two-way ANOVA, two-sided)

*P*_ethnicity × __weight gain_ ethnicity × weight gain intervention interaction (two-way ANOVA, two-sided)

*P_baseline value × __weight gain_ ethnicity × weight gain intervention interaction (two-way ANCOVA, adjusted for baseline values, two-sided)

Supplementary Table 2 shows subcutaneous abdominal adipocyte gene expression data at baseline and in response to weight gain in the two groups. Of the 25 adipocyte genes evaluated, 11 were expressed differently between SA and WE at baseline (all *P* < 0.05). These changes were consistent with SAs having lower expression of genes in the lipid metabolism pathway (lower expression of *ADIPOQ* (by 24%), *APOE*, (by 44%) and *LPL* (by 16%)), and higher expression of *LEP* (by 76%) likely reflecting their higher fat mass. SAs also had lower expression of several insulin signalling pathway genes (*CIDEA* (by 49%), *ESR1* (by 34%), *GHR* (by 42%), *INSR* (by 30%) and *SIRT1* (by 25%)), and higher expression of the negative regulator of adipocyte differentiation *TGFB1* (by 51%) and the tissue stress response/inflammation gene *TNF* (by 160%). Although expression of several genes (*APOE*, *LEP*, *SREBF1*, *CIDEA*, *GHR*, *INSR*, *PLIN2*, *SIRT1*, *TCF7L2*, *TLR2*) changed in response to weight gain overall, only expression of the lipid metabolism pathway gene *SREBF1* changed differently in response to weight gain between the two ethnic groups (*P* = 0.04 for ethnicity × weight gain interaction) with a 54% increase in expression in SAs compared with an 11% increase in WEs. This ethnicity × weight gain interaction in *SREBF1* expression was strengthened after adjustment for *SREBF1* expression at baseline (*P* = 0.004). There were several significant univariable associations between abdominal adipocyte gene expression and adipocyte size variables (Supplementary Table 3).

### Correlates of metabolic responses change with weight gain

Six variables related to insulin sensitivity/insulin resistance and insulin secretion/beta cell function changed differently in response to weight gain between SA and WE: fasting insulin, postprandial insulin, HOMA_IR_, the Matsuda insulin sensitivity index, insulin AUC/glucose AUC and ISSI-2. Significant univariable correlates of changes in these six metabolic responses are shown in Table 4. As expected, ethnicity was a significant correlate of change in all six indices. In terms of body composition variables, changes in fasting insulin concentrations and HOMA_IR_ were significantly (positively) correlated with baseline values of visceral fat and upper-body adiposity, whereas changes in postprandial insulin concentrations were significantly correlated with baseline ASAT (positively) and whole-body lean tissue (negatively). Given that SAs had significantly higher ASAT and lower lean body tissue, as well as numerically (by ~27%) but not significantly higher visceral fat, at baseline than WEs, these relationships are consistent with differences in baseline body composition contributing to the ethnic differences in changes in insulin sensitivity/insulin resistance variables with weight gain. Changes in Matsuda insulin sensitivity index and ISSI-2 were significantly correlated with change in visceral fat, but these associations were in the opposite direction to that which might have been anticipated—a larger increase in visceral fat was associated with smaller decreases in Matsuda insulin sensitivity index and ISSI-2. However, this is consistent with the larger increase in visceral fat with weight gain in WE. There were no other significant associations of baseline values or changes in any other body composition variables (including liver and muscle fat fractions) with changes in indices of insulin sensitivity/insulin resistance or insulin secretion/beta cell function.

**Table 4:** Significant univariable correlates of changes in indices of insulin sensitivity/insulin resistance with weight gain

	Univariable correlate	*r*	95% CI	*P*
**Change in fasting insulin concentration**	Ethnicity	0.385	(0.059, 0.637)	0.02
	Baseline visceral fat	0.482	(0.167, 0.708)	0.004
	Baseline upper-body adipose tissue	0.424	(0.094, 0.670)	0.01
	Baseline proportion of small adipocytes	−0.454	(−0.700, −0.112)	0.01
	Baseline volume of small adipocytes	−0.421	(−0.678, −0.071)	0.02
	Baseline proportion of medium adipocytes	0.411	(0.059, 0.672)	0.01
	Baseline volume of medium adipocytes	0.430	(0.082, 0.684)	0.02
	Change in proportion of very small adipocytes	0.427	(0.079, 0.682)	0.02
	Change in volume of very small adipocytes	0.488	(0.155, 0.721)	0.006
**Change in postprandial insulin concentration**	Ethnicity	0.413	(0.092, 0.656)	0.01
	Baseline ASAT	0.403	(0.070, 0.656)	0.02
	Baseline whole-body lean tissue	−0.370	(−0.633, −0.030)	0.03
	Baseline mean adipocyte diameter	0.573	(0.268, 0.774)	0.001
	Baseline adipocyte volume	0.621	(0.336, 0.802)	<0.0001
	Baseline proportion of small adipocytes	−0.560	(−0.766, −0.250)	0.001
	Baseline volume of small adipocytes	−0.663	(−0.866, −0.398)	<0.0001
	Baseline proportion of large adipocytes	0.701	(0.456, 0.847)	<0.0001
	Baseline volume of large adipocytes	0.644	(0.369, 0.815)	<0.0001
**Change in HOMA**_**IR**_	Ethnicity	0.410	(0.089, 0.654)	0.01
	Baseline visceral fat	0.382	(0.044, 0.641)	0.03
	Baseline upper-body adipose tissue	0.352	(0.010, 0.620)	0.05
	Baseline proportion of small adipocytes	−0.441	(−0.691, −0.096)	0.02
	Baseline volume of small adipocytes	−0.416	(−0.675, −0.066)	0.02
	Baseline proportion of medium adipocytes	0.389	(0.033, 0.657)	0.03
	Baseline volume of medium adipocytes	0.411	(0.060, 0.672)	0.02
	Change in volume of very small adipocytes	0.412	(0.060, 0.672)	0.02
**Change in Matsuda insulin sensitivity index**	Ethnicity	−0.393	(−0.642, −0.068)	0.02
	Change in visceral fat	0.348	(0.006, 0.618)	0.05
	Baseline visceral fat	0.368	(0.028, 0.631)	0.04
	Baseline ASAT	0.405	(0.071, 0.657)	0.02
	Baseline whole-body lean tissue	−0.378	(−0.639, −0.040)	0.03
	Baseline whole-body adipose tissue	0.395	(0.060, 0.650)	0.02
	Baseline upper-body adipose tissue	0.413	(0.082, 0.663)	0.02
	Baseline mean adipocyte diameter	0.603	(0.310, 0.971)	<0.0001
	Baseline adipocyte volume	0.621	(0.335, 0.802)	<0.0001
	Baseline proportion of small adipocytes	−0.592	(−0.785, −0.294)	0.001
	Baseline volume of small adipocytes	−0.676	(−0.833, −0.418)	<0.0001
	Baseline proportion of large adipocytes	0.683	(0.428, 0.837)	<0.0001
	Baseline volume of large adipocytes	0.620	(0.335, 0.801)	<0.0001
**Change in ISSI-2**	Ethnicity	−0.410	(−0.654, −0.089)	0.01
	Change in visceral fat	0.391	(0.055, 0.647)	0.03

*r* and *P* values obtained from two-sided, univariable linear regression, without adjustment for multiple comparisons. CI, confidence interval.

**Fig. 4:**
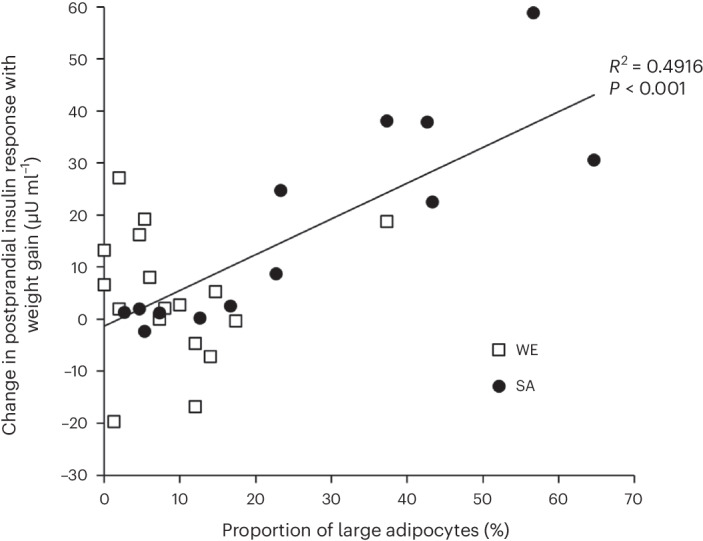
Scatterplot of the relationship between change in postprandial insulin response with weight gain and proportion of large subcutaneous abdominal adipocytes at baseline. *R*^2^ and *P* values obtained from two-sided linear regression. Large subcutaneous abdominal adipocytes are >90 μm diameter. *n* = 13 SAs, *n* = 17 WEs). Source data

In terms of adipocyte morphology, changes in fasting insulin concentration and HOMA_IR_ were negatively correlated with the proportion and volume of small adipocytes and positively correlated with the proportion and volume of medium adipocytes. Thus, individuals whose distribution of adipocytes was skewed towards having more small adipocytes at baseline experienced smaller adverse changes in fasting insulin and HOMA_IR_ with weight gain. A broadly similar pattern was seen when considering correlates of the change in the postprandial insulin response and insulin AUC/glucose AUC ratio, with the proportion and volume of small adipocytes being negatively correlated, and the proportion and volume of large adipocytes being positively correlated, with change in the response with weight gain. The change in fasting insulin concentration was associated with the change in the proportion and volume of very small adipocytes with weight gain. Similarly, the change in HOMA_IR_ was significantly associated with the change in the volume of very small adipocytes with weight gain. These associations are consistent with ethnic differences in adipocyte morphology contributing to the ethnic differences in the changes in insulin sensitivity/resistance indices with weight gain. SAs had a smaller proportion and volume of small adipocytes at baseline, a larger proportion and volume of large adipocytes and experienced a smaller decrease in the proportion and volume of very small adipocytes with weight gain than WEs. Adipocyte morphology variables were highly correlated with each other and inclusion of multiple indices of adipocyte morphology in a single multivariable model did not appreciably alter the variance in the change in the metabolic outcome with weight gain explained compared with univariable models. For example, a univariable model including only a proportion of large adipocytes at baseline explained 49.1% (that is, 0.701^2^) of the variance in the change in postprandial insulin response (Fig. 4), whereas a multivariable model including all significant adipocyte morphology variables from univariable models (that is, baseline mean adipocyte diameter, baseline adipocyte volume, baseline proportion and volume of small adipocytes, and baseline proportion and volume of large adipocytes) explained 54.3% of the variance.

To understand the extent to which correlations between indices of adipocyte size and changes in metabolic outcomes were independent of other body composition variables, we ran multivariable models including the adipocyte size variable most strongly associated with change in the metabolic outcome together with the body composition variables significantly associated with change in the metabolic outcome in univariable analyses in the same model. For the change in fasting insulin concentration, change in volume of very small adipocytes explained 23.8% of the variance in a univariable model, whereas a multivariable model including change in volume of very small adipocytes, together with baseline visceral fat and baseline ASAT, explained 28.4% of the variance, with change in volume of very small adipocytes being the only significant explanatory variable in the model (*P* = 0.04; *P* for visceral fat = 0.20; *P* for ASAT = 0.94). For change in the postprandial insulin response, 49.1% was explained by baseline proportion of large adipocytes alone, and 52.3% was explained by a multivariable model including baseline proportion of large adipocytes, baseline ASAT and baseline whole-body lean tissue, with baseline proportion of large adipocytes being the only significant explanatory variable (*P* = 0.003; *P* for ASAT = 0.47; *P* for lean tissue = 0.23). Similarly, for change in insulin AUC/glucose AUC ratio, baseline proportion of large adipocytes alone explained 46.6% of the variance, and baseline proportion of large adipocytes, together with baseline visceral fat, baseline ASAT, baseline whole-body lean tissue, baseline whole-body adipose tissue and baseline upper-body adipose tissue, explained 52.3% of the variance, with baseline proportion of large adipocytes being the only significant explanatory variable (*P* = 0.01; *P* for visceral fat = 0.84, *P* for ASAT = 0.60; *P* for lean tissue = 0.23, *P* for whole-body adipose tissue = 0.95; *P* for upper-body adipose tissue = 0.92). For change in HOMA_IR_, baseline proportion of small adipocytes explained 19.4% of the variance in a univariable model, and a multivariable model including baseline visceral fat and baseline ASAT explained 23.9% of the variance. However, in this multivariable model, no individual explanatory variable was statistically significant (*P* for small adipocytes = 0.10; *P* for visceral fat = 0.31; *P* for ASAT = 0.44). Thus, overall, these data suggest that indices of adipocyte size explained changes in metabolic variables with weight gain, independently of body composition variables.

## Discussion

Our main finding was that the metabolic consequences of modest weight gain in normoglycaemic men without overweight or obesity differed substantially between men of SA compared with WE descent. While fasting and postprandial glucose responses did not change with weight gain within either group, changes in several indices of insulin sensitivity/insulin resistance and insulin secretion/beta cell function differed markedly between ethnic groups. In men with a starting BMI of ~22 kg m^−2^ in their early 20s, a ~4.5-kg (~1.4 kg m^−2^ BMI) increase in body mass substantially reduced insulin sensitivity in men of SA origin (38% decrease in insulin sensitivity index) but only resulted in a small decrease in insulin sensitivity in men of WE origin (7% decrease), with the pattern of changes in fasting and postprandial insulin concentrations, HOMA_IR_ and estimated insulin secretion and beta cell function following the same patterns. These data suggest that SA men have less ‘metabolic buffering capacity’ than WE men, such that relatively modest weight gain, even within the normal BMI range, can lead to substantial metabolic dysfunction.

At baseline, SA participants had greater adiposity and lower lean mass than their WE peers, consistent with previous reports^10–13^. However, although SA men had more ASAT, visceral fat did not differ significantly between ethnic groups, in agreement with a recent meta-analysis^14^. Also consistent with meta-analysis data^14^, liver fat was higher in the SA group. Although accelerometer-measured physical activity did not differ between groups, cardiorespiratory fitness was ~14% lower in the SA group, in agreement with other reports^10–12^. Baseline postprandial insulin concentrations were 62% (*P* = 0.006) higher in SAs than WEs, although fasting glucose and insulin concentrations, and postprandial glucose concentrations, did not differ significantly at baseline between the ethnic groups. As a consequence of this pattern of ethnic difference in fasting and postprandial glucose and insulin concentrations, HOMA_IR_ (which considers fasting glucose and insulin)^15^ and the Matsuda insulin sensitivity index (which equally weights fasting glucose, postprandial glucose, fasting insulin and postprandial insulin)^16^ did not differ significantly between groups at baseline, and ethnic differences in these measures were only revealed in response to weight gain. This metabolic pattern is similar to our earlier cross-sectional comparisons between young, lean (age ~26 years, BMI ~23 kg m^−2^) SA and WE men, where we reported higher postprandial insulin concentrations in SAs, but similar fasting insulin, fasting glucose and postprandial glucose concentrations in the two ethnic groups^11^. Similarly, Bakker et al. found no difference in fasting insulin concentrations at baseline between young (age 22 years, BMI ~21–22 kg m^−2^) SA and WE men^8^, although, concordant with the present findings, fasting insulin concentrations increased (and insulin sensitivity measured by the euglycaemic–hyperinsulinaemic clamp decreased) substantially more in SAs than WEs in response to 5 days of overfeeding with a high-fat diet. These combined findings show an augmented adverse metabolic response in young, lean SA men in response to an overfeeding challenge. However, the studies differ in one important respect. Bakker et al.^8^ examined the acute effect of short-term positive energy balance without substantial weight gain (participants only gained ~0.5 kg over the intervention period), whereas we investigated the effects of sustained weight gain (~4.5 kg) on metabolic responses in the absence of acute positive energy balance, as we provided participants with identical weight-neutral diets for the 3 days preceding the baseline and post-weight-gain measurements. The present findings are, therefore, analogous to the metabolic state of someone who has gained weight and is now maintaining their new higher weight. The current findings thus extend the earlier work^8^ by showing that weight gain per se, independent of acute dietary changes, leads to a greater adverse metabolic response in young SA men, consistent with the steeper increase in type 2 diabetes risk with increasing BMI in this group^2,3^.

Changes in weight, BMI, waist circumference and whole-, upper- and lower-body adiposity in response to the weight-gain intervention did not differ between ethnic groups. Similarly, there was no difference between groups in changes in ASAT, and no changes in liver or muscle fat were observed. Visceral fat increased more in WEs, such that post-weight-gain visceral fat volumes were similar between the two ethnic groups. Thus, the baseline differences between groups in several adiposity variables did not translate to marked differences in the changes in these variables with weight gain. This may be due to the relatively modest increase in adiposity induced by the intervention, together with measurement errors associated with detecting these changes, limiting the power to detect any differential response between ethnic groups. It is, however, noteworthy that the WE men experienced a more than threefold larger increase in lean tissue in response to weight gain than SA men (2.4 l versus 0.7 l). This anabolic response to weight gain has been observed previously^17,18^ and may act to buffer some of the associated adverse metabolic consequences^19–21^. Our data suggest a degree of anabolic resistance in SA men^13,22–24^, and although the change in lean tissue was not significantly correlated with the change in the postprandial insulin response, this could conceivably reflect lack of statistical power due to measurement error in picking up changes in lean mass in the order of 1–5%. We have recently shown that the effect of a resistance exercise programme on upper-body strength was lower in SA than WE men, although changes in lean mass were similar, and that changes in total, subcutaneous and visceral fat were less favourable in SA men^10^. This suggests that ethnic differences may exist in the proportion of lean and fat mass changes in response to intervention. This may be, at least in part, a consequence of body composition differences at baseline, as in mathematical models of changes in fat and lean mass in response to dietary-induced weight change, higher initial fat mass predicts a larger relative change in body fat^25^. Further studies are needed to confirm whether this anabolic resistance with weight gain is a consistent finding in SA men and is metabolically detrimental.

SAs had substantially larger adipocytes than WEs at baseline, with mean adipocyte diameter and volume being 18% and 76% larger, respectively, and had a larger proportion of large adipocytes (26.2% versus 9.1%) and smaller proportion of small adipocytes (37.1% versus 60.0%) than WEs, consistent with previous findings^5,6,26^. The population of small adipocytes described here have a diameter of 31–70 µm and are consistent with a population of small, immature adipocytes described by McLaughlin and colleagues^27^. These cells are too large to be pre-adipocytes but instead form a population of immature adipocytes that may be recruited to form fully differentiated, mature adipocytes that store additional fat in response to calorie excess. This population of adipocytes, which act as a ‘buffer’ for excess lipid storage requirements, may be available to WEs, whereas in SAs this buffering capacity may already have been exceeded, despite their low BMI, leading to greater adverse consequences of weight gain. This suggestion is supported by the observation that the proportion and/or volume of small adipocytes was significantly negatively associated with the magnitude of increases in fasting and postprandial insulin concentrations, HOMA_IR_ and estimated insulin secretion, in response to weight gain; in other words, those with more small adipocytes were protected from the adverse metabolic effects of gaining weight. The greater proportion of large adipocytes observed in SAs is likely to be consequence of a relative inability to recruit small immature adipocytes^26^, and we observed that the baseline proportion and volume of large adipocytes was strongly positively associated with the change in the postprandial insulin response and estimated insulin secretion with weight gain. These data suggest that ethnic differences in adipocyte morphology, with SA men having fewer small and more large adipocytes, may be an important driver of their greater adverse metabolic change in response to weight gain.

Further research is needed to increase our understanding of why, for a given BMI, SA men have fewer small and more hypertrophic adipocytes, but our exploratory investigations of adipocyte gene expression may provide some initial insights. At baseline, SAs had lower adipocyte expression of genes involved in triglyceride uptake, turnover and storage; and lower expression of genes involved in insulin signalling, and expression of several of these genes were significantly correlated with adipocyte size variables. This is consistent with previous observations that markers of lipogenesis and insulin sensitivity are inversely related to adipocyte size^28^ and that impaired adipocyte lipogenesis is associated with systemic insulin resistance^29^.

There was considerable interindividual variability in the changes in adipocyte size and distribution with weight gain. In this context, no significant changes were observed in adipocyte size variables in response to the weight gain intervention overall. This may be due to the relatively modest degree of weight gain achieved in the intervention—albeit sufficient to decrease insulin sensitivity—and larger numbers of participants (or more substantial weight gain) may be needed to provide sufficient power to detect clear effects. However, it is notable that there was a significant ethnicity × weight gain interaction for the changes in proportion and volume of very small adipocytes (after adjustment for the baseline values), with WEs experiencing a greater decrease with weight gain in this adipocyte pool than SAs. This implies greater lipogenesis in these very small adipocytes with weight gain in WEs leading to them increasing in size beyond the ≤30 μm range. The change in proportion and volume of very small adipocytes was associated with the change in fasting insulin concentration and HOMA_IR_ with weight gain. Thus, ethnic differences in both adipocyte morphology at baseline and the changes in adipocyte morphology with weight gain were associated with the greater adverse metabolic changes with weight gain in SAs.

It was interesting to observe that, overall, there were substantial changes in adipocyte expression of several lipid metabolism, insulin signalling and tissue stress response genes with weight gain. This occurred without significant overall changes in adipocyte size with weight gain, suggesting that functional changes in adipose tissue in response to weight gain may precede overt morphological changes, although significant correlations between changes in adipocyte gene expression and changes in adipocyte size variables were observed for some genes. There was a significant ethnicity × weight gain interaction only for the change in *SREBF1* expression; this was robust to adjustment for multiple comparisons. While insulin can directly activate *SREBF1* and promote lipogenesis, it has also been proposed^30^ that a homeostatic feedback mechanism involving *INSIG1* regulation of SREBP1 (the protein product of the *SREBF1* gene) may be responsible for overriding the suppression of lipogenesis in insulin-resistant states. In this scenario, a decrease in *INSIG1* expression leads to an increase in SREBP1 maturation, increased lipogenesis and facilitation of fat storage despite insulin resistance. The suggested purpose of resetting this regulatory feedback loop is to preserve the synthesis of key functional lipids and maintain adipose tissue lipid homeostasis in the face of lipid overload, and these mechanisms have been observed in humans with obesity and morbid obesity^30^. It is not clear to what extent this post-translational adaptation may impact on *SREBF1* expression, but one might speculate that SA men reach adipocyte lipid overload and initiate a change in homeostatic set point at a lower BMI than their WE counterparts. It is interesting to note that others have found that adipocyte hypertrophy, and higher *SREBF1* expression, is associated with lower VAT relative to total abdominal (VAT plus ASAT) adipose tissue depots in adolescents with obesity^31^, which is consistent with the smaller increase in VAT with weight gain observed in SA participants in the present study. However, these observations and interpretations should be considered exploratory and require independent confirmation in a future study.

A key strength of this study is its longitudinal design, which facilitated greater insights into ethnic differences in response to weight gain than could be obtained from the existing cross-sectional studies. The intervention was well controlled, and the same degree of weight gain was achieved in both groups. However, we were unable to reach our original recruitment target, which limited power to further explore the correlates of differential metabolic response to weight gain. The study was also limited to young, lean men, who we chose as the participant group in this initial study for two main reasons. The first was ethical. The risk of inducing long-term adverse health outcomes with modest weight gain would be higher in groups who were older, were more insulin resistant or had greater baseline adiposity. The second was practical. We studied men rather than women because of the additional complication of controlling for menstrual cycle in an already challenging protocol—there is clear evidence that metabolic responses can differ substantially between follicular and luteal phases^32^—and because women need to gain more weight than men to develop similar metabolic perturbances^33^. It is notable that most invasive studies investigating mechanisms underpinning the increased type 2 diabetes risk in SAs have, to date, focused on men^5,8,11^. This is a clear limitation to the field, which will require redress in future studies, because although SA women, like SA men, experience larger increases in type 2 diabetes risk with increasing BMI than their WE counterparts, the biological mechanisms responsible are not necessarily identical between the sexes. For example, we recently reported in a meta-analysis that, in contrast to observations in men, SA women do not carry more ASAT than their WE counterparts (although SAs of both sexes have higher levels of liver fat)^7^.

In conclusion, this study demonstrated that modest weight gain led to substantially greater adverse metabolic consequences in SA men without overweight or obesity than their WE peers. Thus, while WE men appear to exhibit a degree of metabolic buffering capacity before weight gain leads to adverse metabolic consequences, this does not appear to be present in SA men. Our data suggests that SAs having a greater proportion of hypertrophic adipocytes, and fewer small adipocytes, contributes to the effect, reinforcing the imperative for prevention of weight gain in this group.

## Methods

### Participants and recruitment

The Glasgow Visceral and Ectopic Fat With Weight Gain in South Asians (GlasVEGAS) study was registered on ClinicalTrials.gov (NCT02399423). Ethical approval was obtained from the University of Glasgow College of Medical, Veterinary and Life Sciences Ethics Committee, and all participants provided written informed consent. The ethics application and study protocol are included in the Supplementary Information. Participants were men (assessed by self-report), aged 18–45 years, with BMI < 25 kg m^2^, who had been weight stable (±2 kg) for >6 months, and were either of WE (self-report of both parents of WE ethnic origin) or SA (self-report of both parents of Indian, Pakistani, Bangladeshi or Sri Lankan ethnic origin) ethnic origin. Participants were recruited via personal contacts and local advertising. Exclusion criteria included diabetes (physician-diagnosed or haemoglobin A1c ≥ 6.5% (48 mmol mol^−1^) on screening), history of cardiovascular disease, regular participation in vigorous physical activity, current smoking, taking drugs or supplements thought to affect carbohydrate or lipid metabolism, or other significant illness that would prevent full participation in the study. The study was undertaken in Glasgow, United Kingdom and participant recruitment occurred between 2015 and 2017. Write-up was delayed by the first author returning to a clinical role after completion of PhD and the effects of the coronavirus disease 2019 pandemic delaying adipocyte gene expression analyses.

### Study design

All participants underwent an overfeeding protocol to induce ~7% (minimum of 5%) gain of body mass over 4–6 weeks. This was achieved by asking participants to eat until they felt more full than usual and providing them with high-energy snacks (ice-cream, chocolate bars, potato crisps, cheese, dried fruit and nuts, sugary drinks) to supplement their usual food intake by ~6.2–8.4 MJ per day (1,500–2,000 kcal per day). Once participants gained the required weight, they were placed on a weight-neutral diet for 3 days before the post-weight-gain assessments were made. Assessments of body fat and fat distribution, adipose tissue morphology, metabolic responses to eating, energy expenditure, dietary intake, physical activity and fitness were undertaken at baseline and after weight gain. Participants were then supported to lose the excess weight gained over the following 12 weeks. All but one participant who completed the weight loss phase were followed up until their weight returned to baseline (Fig. 1). However, a large number of participants, particularly in the SA group, declined to undertake post-weight-loss assessments, which meant that power to robustly detect any ethnic differences in responses in the weight loss phase of the study were limited (32% power to detect a 1-s.d. difference between groups). Thus, data are presented for the weight gain phase only.

### Measurement of body composition

Height, weight and waist circumference were measured at baseline and following weight gain. Magnetic resonance imaging (MRI) was performed using 3.0-Tesla MRI scanner (Magnetom, Siemens) using a dual-echo 50-min Dixon Vibe protocol from head to feet, which provided water- and fat-separated volumetric data^34^. Body composition analysed included whole-body lean tissue; upper-body, lower-body and whole-body adipose tissue; and abdominal subcutaneous adipose tissue and VAT. Arms were not included in the whole-body or upper-body MRI body composition measurements. ^1^H magnetic resonance spectroscopy was undertaken to measure liver fat and quadriceps intramuscular triglyceride levels. Spectra were analysed using jMRUI software and the AMARES algorithm^35,36^.

### Metabolic testing

For 3 days before baseline and post-weight-gain metabolic assessments, all participants’ food was provided, with the amount calculated to ensure a stable body weight during this period. Resting metabolic rate was calculated via indirect calorimetry using a ventilated hood (Quark CPET metabolic cart, COSMED)^37^ and energy intake was calculated as 1.55 times resting metabolic rate, in line with estimated daily energy requirements for a lightly active man^38^. ‘Westernized’ foods were provided in these standardized diets with macronutrient proportions of 35–40% fat, 45–50% carbohydrate and 12–17% protein. During these 3-day periods, participants were advised not to exercise or to eat any additional food. Participants monitored their body weight daily to ensure weight remained stable (within 0.5 kg) during these 3-day periods. Physical activity was monitored using hip-worn accelerometers (Actigraph 3TX+, Actigraph) worn during all waking hours for 7 days before each metabolic test day.

On metabolic test days, participants arrived after an overnight fast. Resting metabolic rate and substrate utilization were measured by indirect calorimetry. Then a ~0.5 g subcutaneous abdominal adipose tissue biopsy sample was taken, under local anaesthetic (10 ml 1% lignocaine), lateral to the umbilicus by liposuction with a 14-gauge biopsy needle (Sterican Single Use Deep Intramuscular Needle, B Braun Medical) attached to a 50 ml Luer Lock sterile syringe pre-filled with 5 ml of sterile saline. An ice pack was applied for 20 min to minimize swelling and bruising. A cannula was inserted into an arm vein and a fasting blood sample taken. To provide a physiologically relevant metabolic challenge representative of daily life, participants were given a standard mixed test meal, comprising a plain bagel (New York Bakery Company) with polyunsaturated fat margarine (Flora original; Unilever) and a meal-replacement drink (Strawberry-flavoured Complan, Complan Foods Limited) made with whole milk, containing 3.34 MJ (~800 kcal), with 37% of energy from fat, 47% from carbohydrate and 17% from protein, which was consumed over 10–15 min. Further blood samples were taken 30, 45, 60, 90, 120, 180, 240 and 300 min postprandially. Plasma samples were analysed for glucose, insulin and triglycerides. Time-averaged areas under the postprandial concentration-versus-time curves (that is, AUC, calculated using the trapezium rule, divided by the duration of the postprandial observation period, to give the average postprandial concentration) were used as summary measures of postprandial metabolic responses. The Matsuda insulin sensitivity index was calculated as a measure of whole-body insulin sensitivity^16^. HOMA_IR_ was calculated from glucose and insulin concentrations in the fasted state^15^. Insulin secretion was estimated as insulin AUC/glucose AUC^39^. The ISSI-2, defined as insulin AUC/glucose AUC multiplied by the Matsuda insulin sensitivity index, was used as an estimate of beta cell function^40^.

### Preparation of adipocytes and adipocyte sizing

Adipose tissue samples were placed in collection buffer (Hank’s Medium 199, Thermo Fisher Scientific, 22350029), and tissue temperature was maintained at 37 °C throughout. Adipocyte cell suspensions were then prepared as described by Rodbell^41^. Five microlitres of adipocyte 90% cytocrit suspension was split into two 5 μl aliquots of Krebs Ringer Hepes buffer on a glass slide, and digital pictures of adipocytes were captured using a ×10 lens under a B×50 microscope by Image-Pro Plus 4.0 (Image-Pro Plus version 4, Media Cybernetics). The intracellular diameters of 150 adipocytes in each sample were measured using ImageJ (version 1.52a, W. Rasband at the National Institutes of Health). Each individual adipocyte diameter was placed in a 10-μm-size band from 0–10 μm to >220 μm. Mid-point values for each 10-μm band were used to calculate mean adipocyte diameter and adipocyte volume (expressed as microlitres per 100 cells). There is no consensus in the literature about thresholds for adipocyte size categories. Based on the size distribution of adipocytes in our cohort, we designated adipocytes of ≤30 μm as very small, 31–70 μm as small, 71–90 μm as medium and >90 μm as large, and calculated the proportion and volume of adipocytes within each size category for each participant at baseline and in response to weight gain.

### Adipocyte gene expression

Total RNA was extracted from 50–100 mg adipocyte cells using RNeasy Lipid Tissue Mini Kit (QIAGEN, 74804). The integrity and purification of isolated RNA was confirmed using a NanoDrop 1000 (Thermo Fisher Scientific). The optical density as a 260/280 nm ratio (1.8–2.0 acceptable purity) and the concentration of RNA was recorded. A DNA-free kit (Thermo Fisher Scientific, AM1906) was used to remove any DNA contamination in the RNA samples. Single-stranded complementary DNA (cDNA) used for quantitative PCR was reverse transcribed from purified RNA using a high-capacity cDNA reverse transcription kit (Thermo Fisher Scientific, 4368813). The quantity of specific cDNA targets was pre-amplified using TaqMan PreAmp master mix (2×; Thermo Fisher Scientific, 4384266). Uniformity of pre-amplification was checked using *CDKN1* according to the manufacturer’s instructions. Ingenuity pathway analysis was used to identify potential adipocyte genes that may differ between SAs and WEs at baseline or in response to weight gain using the terms ‘insulin resistance’, ‘weight gain’, ‘weight loss’, ‘size of adipocytes’ and ‘adipocyte differentiation’ from the Ingenuity Pathway Analysis database^42^. Twenty-five genes related to lipid metabolism (*ADIPOQ*, *APOE*, *CYP19A1*, *KLF14*, *LDLR*, *LEP*, *LPL*, *SREBF1*), insulin signalling (*CASP1*, *CIDEA*, *ESR1*, *GHR*, *INSR*, *PIK3R1*, *PLIN2*, *SIRT1*), adipocyte differentiation (*BSCL2*, *EPAS1*, *HOXC13*, *PPARG*, *TGFB1*), and tissue stress response or inflammation (*HIF1A*, *TCF7L2*, *TNF*, *TLR2*) were selected for study (see Supplementary Table 1 for details of gene functions and assay IDs). The RT–PCR reaction mix was made up for each target gene using 1.25 µl target TaqMan Gene Expression Assay and 12.5 µl TaqMan universal PCR master mix (Thermo Fisher Scientific, 4304437). Diluted pre-amplified cDNA (6.25 µl) was mixed with each PCR reaction mix in duplicate in a MicroAmp fast optical 96-well reaction plate (Thermo Fisher Scientific) and underwent PCR according to the following programme: 50 °C for 2 min, 95 °C for 10 min, then 40× of 95 °C for 15 s and 60 °C for 1 min (StepOnePlus, Real-Time PCR system). *PPIA* was used as the reference gene^43^, and relative expression to *PPIA* of each gene was calculated as: Relative expression to *PPIA* (%) = 2^−(Ct target gene – Ct PPIA) ^× 100%.

### Assessment of cardiorespiratory fitness

Maximal oxygen uptake was assessed using a modified Taylor incremental uphill walking protocol with a treadmill speed of 5.5 km h^−1^ and gradient increasing by 3% every 2 min^44^. Oxygen uptake was determined from 1-min expired air samples taken continuously and heart rate was monitored by short-range telemetry. Achievement of maximal oxygen uptake was verified by volitional exhaustion together with a rate of perceived exertion of 19–20, a respiratory exchange ratio of >1.15 and heart rate within ten beats of the age-predicted maximum.

### Power calculation and statistical analysis

The sample size was based on the power to detect a 1-s.d. difference for change in outcomes of interest with weight gain between groups, based on the typically ≥1-s.d. difference in postprandial insulin response, fat mass and adipocyte size observed in previous comparisons between normoglycaemic SA and WE adults^5,11^. Twenty-three participants per group provided 90% power to detect this magnitude of difference, with 80% power provided with 17 per group. To achieve 23 completers per group, allowing for drop out, we aimed to recruit 30 participants per group (that is, 30% over-recruitment). Our final achieved power to detect a 1-s.d. difference with 21 WE and 14 SA participants completing the study was 80.3%.

Statistical analysis was undertaken using Statistica (version 10.0, StatSoft) and Minitab (version 20.0, Minitab, State College). Data distribution was assumed to be normal, but this was not formally tested. Differences between ethnic groups at baseline were analysed using unpaired *t*-tests. The effects of weight gain (main effect of weight gain) and differences between ethnic groups in changes due to weight gain (weight gain × ethnicity interaction) were analysed by two-way ANOVA (ethnicity × time), with repeated-measures analysis on the time factor (baseline, after intervention). To assess whether ethnic differences in responses to weight gain were independent of any ethnic differences in baseline values, two-way ANCOVAs were performed, with baseline values included as a covariate in the models. To help understand factors contributing to the ethnic differences in the changes in fasting insulin concentrations, the postprandial insulin response, HOMA_IR_, the Matsuda insulin sensitivity index, insulin AUC/glucose AUC and ISSI-2 with weight gain (change in glucose and triglyceride responses with weight gain did not differ between ethnic groups), univariable and multivariable linear regressions between the change in these variables with weight gain, with body composition variables, fitness and physical activity variables and adipocyte size variables were explored. Univariable associations between adipocyte gene expression and adipocyte size variables were also investigated in exploratory analyses. All tests were two-sided, and statistical significance was accepted at a nominal value of *P* < 0.05. In this basic human experimental study, our primary aims were around ethnic differences in responses to weight gain in three ‘families’ of data—anthropometric/body composition (10 outcomes, excluding change in weight and BMI, which is the same in both groups, by design), metabolic (12 outcomes) and adipocyte morphology (10 outcomes). We also reported data on changes in cardiorespiratory fitness and physical activity (8 outcomes) and undertook an exploratory analysis of ethnic differences in expression of adipocyte genes related to lipid metabolism, adipocyte differentiation, tissue stress response and inflammation (25 outcomes). To reduce the familywise risk of type I statistical error, we used the method described by Tukey et al.^45^ (Bonferroni adjustment is considered too conservative when there are multiple high-correlated outcomes as is the case in the present study^45^), to adjust the *P* value for statistical significance from 0.05 to 0.02 for body composition, metabolic, adipocyte morphology and fitness/physical activity outcomes (assuming 12 outcomes in each family) and to 0.01 for adipocyte gene expression outcomes (25 outcomes). Biochemical analyses and adipocyte sizing and gene expression analyses were blinded. Data collection and statistical analysis were not performed blind to the conditions of the experiments. All available data points were included in the analysis.

### Reporting summary

Further information on research design is available in the Nature Portfolio Reporting Summary linked to this article.

### Supplementary information

Supplementary Information Supplementary Tables 1–3 and GlasVEGAS ethics application and Study protocol.

Reporting Summary

### Source data

Source Data Figs. 2–4 Statistical source data.

## Data Availability

Anonymized data generated during and analysed during the current study will be available on request. The full dataset contains several indirect identifiers so unfortunately cannot be shared publicly (beyond the aggregate data presented in the paper) in line with current guidance^46^. Source data are provided with this paper.
