# Supplementary material for: Weight gain leads to greater adverse metabolic responses in South Asian compared with white European men: the GlasVEGAS study
Source: Nat Metab. 2024 Aug 16;6(8):1632–45. doi: 10.1038/s42255-024-01101-z (PMC11349579; doi:10.1038/s42255-024-01101-z)
Supplement: Supplementary file 1 — Supplementary Tables 1–3 and GlasVEGAS ethics application and Study protocol. [file 42255_2024_1101_MOESM1_ESM.pdf]

nature metabolism

Supplementary information

https://doi.org/10.1038/s42255-024-01101-z

Weight gain leads to greater adverse metabolic responses in South Asian compared with white European men: the GlasVEGAS study

In the format provided by the authors and unedited

SUPPLEMENTARY MATERIAL

TABLE OF CONTENTS

**Supplementary Table 1**	**page 2**
**Supplementary Table 2**	**page 6**
**Supplementary Table 3**	**page 8**
**Supplementary Materials References**	**page 13**
**Glasvegas Ethics Application and Study Protocol**	**page 15**

Supplementary Table 1. Function of candidate genes by different pathways in adipocytes.

	Gene symbol	Assay ID	Function of gene
**Lipid metabolism pathway**	*ADIPOQ*	Hs00605917_m1	Codes for adiponectin, an adipokine. End differentiation marker for adipose tissue. Adiponectin can also increase lipid oxidation and decrease the secretion of free fatty acid (FFA) by activating the AMP-activated protein kinase (AMPK) complex in adipocytes. Adiponectin can reduce plasma glucose and increase insulin sensitivity in the whole body. [1]
	APOE	Hs00171168_m1	Codes for apolipoprotein E. Stimulates adipocyte triglyceride turnover and involved in lipoprotein uptake [2,3]. Apolipoprotein E is a major binding protein for cholesterol transporters including LDL, HDL and VLDL [2].
	CYP19A1	Hs00903411_m1	Codes for aromatase, which converts a class of hormones called androgens. In males, aromatase is most active in adipocytes. Androgen regulates lipid and cholesterol metabolism by stimulating the activities of LPL and HSL [4].
	KLF14	Hs00370951_s1	Codes for Kruppel-like factor 14. Strongly associated with low HDL cholesterol levels, high TG levels, risk of type 2 diabetes mellitus [5]. It is master regulator of gene expression in subcutaneous adipocytes and the mechanism is not exclusively understood.
	LDLR	Hs00181192_m1	Codes for the LDL receptor. Bind remnants of chylomicrons and LDL, mediates lipid transport [6].
	LEP	Hs00174877_m1	Codes for leptin, an adipokine. It increases lipid oxidation and decreases the secretion of FFA by activating the AMPK complex in adipocytes. Negatively correlates with total body fat storage by inhibiting food intake and decreases adipocyte sensitivity to insulin thereby inhibiting lipid accumulation [7].
	LPL	Hs00173425_m1	Codes for lipoprotein lipase. It is an enzyme that hydrolyses circulating TG so that it generates FA used in the synthesis of TG in lipid droplets. Essential for lipid uptake and storage [8].
	SREBF1	Hs01088691_m1	Codes for Sterol Regulatory Element-Binding Transcription Factor 1. SREBP1c is the isoform mainly expressed in liver, muscle and fat and regulates genes required for glucose metabolism and fatty acid and lipid production. Its expression is induced by insulin and SREBP1c plays a major role in promoting liver lipogenesis.

**Insulin signalling pathway**	*CASP1*	Hs00354836_m1	Codes for Caspase 1. Lipolysis stimulates the expression of *CASP1*, which upregulates the secreting agents that promote pyroptosis, resulting in the release of the inflammatory factor TNFα. CASP1 is upregulated during adipogenesis and directs adipocytes into a more insulin resistant phenotype [9].
	CIDEA	Hs00154455_m1	Codes for cell death-inducing DFFA-like effector A. Lipid droplet associated protein and positively correlated with insulin sensitivity [10].
	ESR1	Hs01046816_m1	Codes for estrogen receptor 1. Estrogen improves adipocyte metabolism and insulin sensitivity by facilitating FFA oxidation in SAT, and in VAT, by improving mitochondria β-oxidation [11,12].
	GHR	Hs00174872_m1	Codes for growth hormone receptor. Activation of growth hormone receptor plays an insulin-like role in increasing glucose transport and FA oxidation, by activation of the PI3K/AKT pathway. Strong regulator of lipid and glucose metabolism and plays a key role the proliferation and differentiation of pre-adipocytes [13,14].
	INSR	Hs00961554_m1	Codes for insulin receptor. A major endocrine hormone receptor involved in the regulation of energy and lipid metabolism.
	PIK3R1	Hs00933163_m1	Codes for phosphatidylinositol 3-kinase. A key kinase in the insulin pathway, downstream of the insulin receptor.
	PLIN2	Hs00605340_m1	Codes for Perilipin 2. Expression of *PLIN2* is insulin sensitive. Perilipin 2 is essential for the formation of lipid droplets in adipocytes to store fat [15]
	SIRT1	Hs01009006_m1	Codes for sirtuin 1. It is the most conserved mammalian NAD+ dependent histone deacetylase. Regulated by insulin, it is a NAD^+-dependent protein that regulates natural aging and other stress-related conditions [16,17].
**Adipocyte differentiation**	*BSCL2*	Hs00949220_m1	Codes for seipin, a multi-pass transmembrane protein. An essential, cell-autonomous regulator of adipogenesis by regulating the formation of lipid droplets through actin cytoskeleton remodelling [18].
	EPAS1	Hs01026149_m1	Codes for endothelial PAS domain protein 1, a transcription factor. Promotes adipocyte differentiation, but the mechanism is unclear [19].
	HOXC13	Hs00600868_m1	Encodes a highly conserved family of homeodomain-containing transcription factors. Strongly associated with body fat distribution, especially in gluteal fat [20,21]. Down regulated by PPARG [22].
	PPARG	Hs01115513_m1	Codes for peroxisome proliferator-activated receptor. Key factor of terminal adipocyte differentiation. Initiates and maintains adipocyte differentiation and maturation by a positive feedback loop with C/EBPα [23].

	TGFB1	Hs00998133_m1	Codes for transforming growth factor beta 1. In MSC, TGFβ inhibits commitment differentiation to pre-adipocytes by inhibiting expression of *PPARG* [24]. In mature adipocytes, TGFβ stimulates TGFBR/SMAD pathways, which activate extracellular matrix to increase focal adhesion kinase (FAK)/AKT signalling, to stimulate lipogenesis pathways and lipid storage [25].
**Tissue stress response/ inflammation**	*HIF1A*	Hs00153153_m1	Codes for hypoxia inducible factor 1 subunit alpha. Increases chronic inflammatory responses in adipocytes in patients with obesity [26]. An important paralog of *EPAS1*.
	TCF7L2	Hs01009044_m1	Codes for transcription factor 7 like 2, inflammatory factor. Regulates adipocyte size, endocrine function, and glucose metabolism by directly regulating genes involved in lipid metabolism (*ADIPOQ*) and insulin signalling pathways (*GLUT4* and *LEP*) [27,28].
	TLR2	Hs00610101_m1	Codes for toll like receptor 2. Plays a fundamental role in pathogen recognition and activation of innate immunity and mediates inflammatory processes in obese adipose tissue. Decreases insulin sensitivity by decreasing the expression of *GLUT4*, and the translocation of GLUT4 [29,30].
	TNF	Hs00174128_m1	Codes for tumour necrosis factor alpha, an inflammatory factor. Promotes inflammatory response and decreases insulin sensitivity in adipocytes [31].

Assay ID: TaqMan® Gene Expression Assays used for RT-qPCR. Abbreviations: ADIPOQ, adiponectin; APOE, apolipoprotein E; BSCL2, Bernardinelli-Seip congenital lipodystrophy type 2 protein; CASP1, caspase 1; CIDEA, cell death inducing DFFA Like effector A; CYP19A1, cytochrome P450 family 19 subfamily A member 1; EPAS1, endothelial PAS domain protein 1; ESR, estrogen receptor; GHR, growth hormone receptor; INSR, insulin receptor; HIF1A, hypoxia inducible factor 1 subunit Alpha; HOXC13, homeobox C13; KLF14, Krueppel-like factor 14; LDLR, low-density lipoprotein receptor; LEP, leptin; LPL, lipoprotein lipase; SREBF1, sterol regulatory element binding transcription factor 1; PIK3R1, phosphoinositide-3-kinase regulatory subunit 1; PLIN2, perilipin 2; SIRT1, sirtuin 1; PPARG, peroxisome proliferator activated receptor gamma; TGFB1, transforming growth factor Beta 1; TCF7L2, transcription factor 7 like 2; TNF, tumour necrosis factor; TLR2, toll like receptor 2.

Supplementary Table 2. Expression of genes of interest in SAT adipocytes in white European and South Asian men at baseline and after weight gain.

		Baseline			Change with weight gain				
	Gene (%expression relative to *PPIA*)	**White European (n = 20)**	**South Asian (n = 14)**	**P** baseline ethnicity	**White European (n = 20)**	**South Asian (n = 14)**	**P** weight gain	**P** ethnicity x weight gain	**P** ethnicity x weight gain (adjusted for baseline)*
Lipid metabolism pathway	*ADIPOQ*	1134.10 ± 72.39	863.54 ± 56.95	0.007	-130.16 ± 89.12	21.21 ± 116.55	0.46	0.30	0.84
	APOE	83.85 ± 12.33	47.37 ± 10.54	0.04	-42.12 ± 9.77	-16.61 ± 6.92	<0.0001	0.06	0.76
	CYP19A1	0.66 ± 0.51	0.08 ± 0.06	0.35	-0.58 ± 0.46	-0.04 ± 0.06	0.28	0.33	0.36
	KLF14	0.89 ± 0.06	0.86 ± 0.1	0.74	-0.03 ± 0.05	0.05 ± 0.09	0.85	0.36	0.34
	LDLR	7.65 ± 1.67	8.70 ± 2.48	0.96	0.37 ± 1.92	-2.67 ± 2.26	0.45	0.32	0.26
	LEP	73.14 ± 8.52	131.72 ± 14.96	0.001	18.97 ± 8.41	41.54 ± 12.1	<0.0001	0.12	0.25
	LPL	458.97 ± 18.99	397.26 ± 21.52	0.03	-12.35 ± 19.92	44.44 ± 34.63	0.40	0.14	0.49
	SREBF1	36.97 ± 3.80	42.18 ± 3.99	0.47	4.24 ± 4.76	22.78 ± 8.03	0.004	0.04	0.004
Insulin signalling pathway	*CASP1*	2.37 ± 0.16	2.93 ± 0.28	0.11	-0.00 ± 0.21	-0.66 ± 0.28	0.07	0.07	0.46
	CIDEA	94.97 ± 9.22	48.42 ± 10.35	0.002	-32.12 ± 9.38	-24.69 ± 10.26	<0.0001	0.60	0.17
	ESR1	7.30 ± 0.64	4.79 ± 0.56	0.008	-1.22 ± 0.92	-1.10 ± 1.11	0.12	0.94	0.06
	GHR	39.10 ± 3.00	22.64 ± 2.86	0.0006	-8.33 ± 3.55	-3.89 ± 2.84	0.02	0.37	0.04
	INSR	12.27 ± 0.84	8.58 ± 0.87	0.003	-2.12 ± 1.14	-1.18 ± 0.85	0.04	0.55	0.18
	PIK3R1	37.86 ± 3.06	31.82 ± 3.14	0.19	1.05 ± 3.63	3.72 ± 3.52	0.37	0.62	0.94
	PLIN2	65.11 ± 7.35	47.68 ± 5.64	0.09	-16.6 ± 6.64	-12.94 ± 7.42	0.006	0.72	0.14
	SIRT1	10.74 ± 0.47	8.08 ± 0.84	0.006	-2.81 ± 0.70	-1.78 ± 0.75	<0.0001	0.33	0.24
Adipocyte differentiation	*BSCL2*	0.12 ± 0.01	0.12 ± 0.02	0.77	-0.03 ± 0.02	-0.02 ± 0.03	0.17	0.80	0.43
	EPAS1	125.86 ± 7.07	132.21 ± 10.12	0.53	-8.98 ± 6.43	-15.44 ± 17.36	0.15	0.70	0.85
	HOXC13	1.14 ± 0.18	0.71 ± 0.12	0.06	0.18 ± 0.41	0.04 ± 0.24	0.68	0.80	0.15
	PPARG	53.94 ± 3.34	46.43 ± 3.31	0.10	-4.25 ± 4.13	-3.94 ± 5.19	0.22	0.96	0.23
	TGFB1	1.82 ± 0.20	2.72 ± 0.41	0.03	0.57 ± 0.30	0.23 ± 0.73	0.26	0.63	0.91
Tissue stress response/ inflammation	*HIF1A*	14.91 ± 0.89	15.23 ± 1	0.83	1.69 ± 1.47	0.45 ± 1.13	0.29	0.54	0.57
	TCF7L2	24.65 ± 1.56	23.17 ± 2.17	0.66	-4.04 ± 2.00	-3.89 ± 1.81	0.009	0.96	0.75
	TLR2	0.83 ± 0.08	0.66 ± 0.12	0.21	-0.27 ± 0.07	-0.22 ± 0.14	0.002	0.72	0.39
	TNF	0.05 ± 0.01	0.13 ± 0.03	0.01	-0.01 ± 0.01	-0.01 ± 0.03	0.65	1.00	0.84

Values are mean ± SEM

P for statistical significance adjusted from 0.05 to 0.01 to control the familywise error rate for multiple comparisons using method described by Tukey et al[32].

$P_{baseline\ ethnicity}$ difference between white Europeans and South Asians at baseline (unpaired t-test, two-sided)

$P_{weight\ gain}$ main effect of weight gain intervention (2-way ANOVA, two-sided)

$P_{ethnicity\ x\ weight\ gain}$ ethnicity x weight gain intervention interaction (2-way ANOVA, two-sided)

*$P_{baseline\ value\ x\ weight\ gain}$ ethnicity x weight gain intervention interaction (2-way ANCOVA, adjusted for baseline values, two-sided)

Abbreviations: ADIPOQ, adiponectin; APOE, apolipoprotein E; BSCL2, Bernardinelli-Seip congenital lipodystrophy type 2 protein; CASP1, caspase 1; CIDEA, cell death inducing DFFA Like effector A; CYP19A1, cytochrome P450 family 19 subfamily A member 1; EPAS1, endothelial PAS domain protein 1; ESR, estrogen receptor; GHR, growth hormone receptor; INSR, insulin receptor; HIF1A, hypoxia inducible factor 1 subunit Alpha; HOXC13, homeobox C13; KLF14, Krueppel-like factor 14; LDLR, low-density lipoprotein receptor; LEP, leptin; LPL, lipoprotein lipase; SREBF1, sterol regulatory element binding transcription factor 1; PIK3R1, phosphoinositide-3-kinase regulatory subunit 1; PLIN2, perilipin 2; SIRT1, sirtuin 1; PPARG, peroxisome proliferator activated receptor gamma; TGFB1, transforming growth factor Beta 1; TCF7L2, transcription factor 7 like 2; TNF, tumour necrosis factor; TLR2, toll like receptor 2.

Supplementary Table 3. Significant uni-variable correlations between adipocyte size and adipocyte gene expression variables

	r	95% CI	P
**Baseline proportion of very small adipocytes**			
Baseline *ADIPOQ*	-0.440	(-0.691, -0.095)	0.02
Baseline *INSR*	-0.365	(-0.641, -0.005)	0.05
**Baseline volume of very small adipocytes**			
Baseline *ADIPOQ*	-0.392	(-0.659, -0.037)	0.03
**Baseline proportion of small adipocytes**			
Baseline *CASP1*	-0.390	(-0.658, -0.034)	0.033
Baseline *INSR*	0.562	(0.253, 0.767)	0.001
Baseline *LEP*	-0.711	(-0.853, -0.471)	<0.0001
Baseline *PPARG*	0.505	(0.177, 0.732)	0.004
Baseline *SIRT1*	0.480	(0.145, 0.716)	0.007
Baseline *TGFB1*	-0.478	(-0.715, -0.142)	0.008
**Baseline volume of small adipocytes**			
Baseline *INSR*	0.479	(0.143, 0.716)	0.007
Baseline *LEP*	-0.683	(-0.837, -0.428)	<0.0001
Baseline *PPARG*	0.512	(0.186, 0.737)	0.004
Baseline *SIRT1*	0.487	(0.154, 0.721)	0.006
Baseline *TGFB1*	-0.478	(-0.715, -0.142)	0.008
**Baseline proportion of medium adipocytes**			
Baseline *HIF1A*	-0.588	(-0.782, -0.288)	0.001
Baseline *HOXC13*	-0.399	(-0.664, -0.046)	0.03
**Baseline volume of medium adipocytes**			
Baseline *HIF1A*	-0.568	(-0.770, -0.261)	0.001

Baseline *HOXC13*	-0.398	(-0.663, -0.046)	0.03
**Baseline proportion of large adipocytes**			
Baseline *INSR*	-0.428	(-0.683, -0.080)	0.02
Baseline *LEP*	0.700	(0.454, 0.847)	<0.0001
Baseline *PPARG*	-0.419	(-0.677, -0.069)	0.02
Baseline *SIRT1*	-0.380	(-0.651, -0.023)	0.04
Baseline *TGFB1*	0.534	(0.215, 0.750)	0.002
**Baseline volume of large adipocytes**			
Baseline *INSR*	-0.380	(-0.651, -0.023)	0.04
Baseline *LEP*	0.608	(0.318, 0.795)	<0.0001
Baseline *TGFB1*	0.554	(0.242, 0.762)	0.001
**Baseline mean adipocyte diameter**			
Baseline *CASP1*	0.43	(0.082, 0.684)	0.02
Baseline *INSR*	-0.453	(-0.699, -0.111)	0.01
Baseline *LEP*	0.655	(0.385, 0.821)	<0.0001
Baseline *PPARG*	-0.397	(-0.663, -0.043)	0.03
Baseline *TGFB1*	0.509	(0.182, 0.734)	0.004
**Baseline adipocyte volume**			
Baseline *INSR*	-0.430	(-0.684, -0.083)	0.02
Baseline *LEP*	0.636	(0.358, 0.811)	<0.0001
Baseline *TGFB1*	0.561	(0.251, 0.766)	0.001
**Change in proportion of very small adipocytes**			
Change in *LDLR*	-0.416	(-0.679, -0.059)	0.03
**Change in volume of very small adipocytes**			

Change in *LDLR*	-0.440	(-0.694, -0.087)	0.02
**Change in proportion of small adipocytes**			
Baseline *TLR2*	0.437	(0.092, 0.689)	0.02
**Change in volume of small adipocytes**			
Baseline *TCF7L2*	0.421	(0.072, 0.679)	0.02
Baseline *TLR2*	0.560	(0.251, 0.766)	0.001
Change in *TLR2*	-0.451	(-0.702, -0.101)	0.01
**Change in proportion of medium adipocytes**			
Baseline *HOXC13*	0.423	(0.074, 0.680)	0.02
Baseline *PPIA*	-0.504	(-0.731, -0.176)	0.005
Change in *BSCL2*	-0.484	(-0.722, -0.143)	0.008
Change in *EPAS1*	-0.605	(-0.795, -0.306)	0.001
Change in *HOXC13*	-0.388	(-0.660, -0.025)	0.04
Change in *KLF14*	-0.403	(-0.670, -0.043)	0.03
Change in *SIRT1*	-0.479	(-0.719, -0.137)	0.009
Change in *TGFB1*	-0.431	(-0.689, -0.076)	0.02
**Change in volume of medium adipocytes**			
Baseline *HOXC13*	0.399	(0.046, 0.664)	0.03
Change in *BSCL2*	-0.465	(-0.711, -0.119)	0.01
Change in *EPAS1*	-0.619	(-0.803, -0.327)	<0.0001
Change in *KLF14*	-0.373	(-0.651, -0.008)	0.05
Change in *LDLR*	0.367	(0.001, 0.647)	0.05
Change in *SIRT1*	-0.451	(-0.701, -0.101)	0.01
Change in *TGFB1*	-0.433	(-0.690, -0.078)	0.02
**Change in proportion of large adipocytes**			

Baseline *KLF14*	-0.429	(-0.684, -0.081)	0.02
Baseline *SIRT1*	-0.450	(-0.697, -0.107)	0.01
Baseline *TCF7L2*	-0.504	(-0.732, -0.176)	0.004
Baseline *TLR2*	-0.492	(-0.724, -0.160)	0.006
Baseline *BSCL2*	-0.386	(-0.655, -0.030)	0.04
Change in *KLF14*	0.447	(0.097, 0.699)	0.02
Change in *TCF7L2*	0.479	(0.137, 0.719)	0.009
Change in *TGFB1*	0.437	(0.084, 0.693)	0.02
Change in *TLR2*	0.461	(0.114, 0.708)	0.01
**Change in volume of large adipocytes**			
Baseline *EPAS1*	-0.397	(-0.663, -0.043)	0.03
Baseline *KLF14*	-0.402	(-0.666, -0.049)	0.03
Baseline *SIRT1*	-0.383	(-0.653, -0.026)	0.04
Baseline *TCF7L2*	-0.445	(-0.694, -0.101)	0.01
Baseline *TGFB1*	-0.422	(-0.679, -0.072)	0.02
Change in *EPAS1*	0.476	(0.132, 0.717)	0.009
Change in *KLF14*	0.514	(0.182, 0.741)	0.004
Change in *TCF7L2*	0.463	(0.116, 0.709)	0.01
Change in *TGFB1*	0.453	(0.103, 0.703)	0.01
**Change in mean adipocyte diameter**			
Baseline *KLF14*	-0.426	(-0.682, -0.078)	0.02
Baseline *TCF7L2*	-0.356	(-0.635, 0.005)	0.05
Baseline *TLR2*	-0.413	(-0.673, -0.063)	0.02
**Change in adipocyte volume**			
Baseline *EPAS1*	-0.362	(-0.639, -0.002)	0.05
Baseline *KLF14*	-0.414	(-0.674, -0.063)	0.02
Baseline *TCF7L2*	-0.407	(-0.669, -0.055)	0.03

Baseline *TGFB1*	-0.441	(-0.691, -0.096)	0.02
Change in *KLF14*	0.424	(0.069, 0.684)	0.02
Change in *TCF7L2*	0.384	(0.021, 0.658)	0.04

r and p-values obtained from two-sided uni-variable linear regression, without adjustment for multiple comparisons.

Supplementary Materials References

1. Kramer, A., Green, J., Pollard, J., Jr. & Tugendreich, S. Causal analysis approaches in Ingenuity Pathway Analysis. *Bioinformatics* **30**, 523-530 (2014).
2. Neville, M.J., Collins, J.M., Gloyn, A.L., McCarthy, M.I. & Karpe, F. Comprehensive human adipose tissue mRNA and microRNA endogenous control selection for quantitative real-time-PCR normalization. *Obesity (Silver Spring)* **19**, 888-892 (2011).
3. Diez, J.J. & Iglesias, P. The role of the novel adipocyte-derived hormone adiponectin in human disease. *European Journal of Endocrinology Eur J Endocrinol* **148**, 293-300 (2003).
4. Huang, Z.H., Reardon, C.A., Getz, G.S., Maeda, N. & Mazzone, T. Selective suppression of adipose tissue apoE expression impacts systemic metabolic phenotype and adipose tissue inflammation. *J Lipid Res* **56**, 215-226 (2015).
5. Huang, Z.H., Reardon, C.A. & Mazzone, T. Endogenous ApoE expression modulates adipocyte triglyceride content and turnover. *Diabetes* **55**, 3394-3402 (2006).
6. Zhao, H.*, et al.* A novel promoter controls Cyp19a1 gene expression in mouse adipose tissue. *Reprod Biol Endocrinol* **7**, 37 (2009).
7. Ding, Q., Gupta Rajat, M., Raghavan, A. & Musunuru, K. Abstract 70: KLF14 is a Novel Regulator of Human Metabolism. *Arteriosclerosis, Thrombosis, and Vascular Biology* **34**, A70-A70 (2014).
8. Hofmann, S.M.*, et al.* Adipocyte LDL receptor-related protein-1 expression modulates postprandial lipid transport and glucose homeostasis in mice. *J Clin Invest* **117**, 3271-3282 (2007).
9. Harris, R.B. Direct and indirect effects of leptin on adipocyte metabolism. *Biochim Biophys Acta* **1842**, 414-423 (2014).
10. Gonzales, A.M. & Orlando, R.A. Role of adipocyte-derived lipoprotein lipase in adipocyte hypertrophy. *Nutr Metab (Lond)* **4**, 22 (2007).
11. Stienstra, R.*, et al.* The inflammasome-mediated caspase-1 activation controls adipocyte differentiation and insulin sensitivity. *Cell Metab* **12**, 593-605 (2010).
12. Abreu-Vieira, G.*, et al.* Cidea improves the metabolic profile through expansion of adipose tissue. *Nat Commun* **6**, 7433 (2015).
13. Zhou, Z.*, et al.* Estrogen receptor α controls metabolism in white and brown adipocytes by regulating Polg1 and mitochondrial remodeling. *Science Translational Medicine* **12**, eaax8096 (2020).
14. Vieira-Potter, V.J., Zidon, T.M. & Padilla, J. Exercise and Estrogen Make Fat Cells "Fit". *Exerc Sport Sci Rev* **43**, 172-178 (2015).
15. Glad, C.A.M.*, et al.* Expression of GHR and Downstream Signaling Genes in Human Adipose Tissue-Relation to Obesity and Weight Change. *J Clin Endocrinol Metab* **104**, 1459-1470 (2019).
16. Ridderstrale, M. Signaling Mechanism for the Insulin-like Effects of Growth Hormone - Another Example of a Classical Hormonal Negative Feedback Loop. *Current drug targets. Immune, endocrine and metabolic disorders* **5**, 79-92 (2005).
17. Li, P., Wang, Y., Zhang, L., Ning, Y. & Zan, L. The Expression Pattern of PLIN2 in Differentiated Adipocytes from Qinchuan Cattle Analysis of Its Protein Structure and Interaction with CGI-58. *Int J Mol Sci* **19**(2018).
18. Hui, X.*, et al.* Adipocyte SIRT1 controls systemic insulin sensitivity by modulating macrophages in adipose tissue. *EMBO Rep* **18**, 645-657 (2017).

19. Salminen, A., Kaarniranta, K. & Kauppinen, A. Crosstalk between Oxidative Stress and SIRT1: Impact on the Aging Process. *Int J Mol Sci* **14**, 3834-3859 (2013).
20. Payne, V.A.*, et al.* The human lipodystrophy gene BSCL2/seipin may be essential for normal adipocyte differentiation. *Diabetes* **57**, 2055-2060 (2008).
21. Shimba, S., Wada, T., Hara, S. & Tezuka, M. EPAS1 promotes adipose differentiation in 3T3-L1 cells. *J Biol Chem* **279**, 40946-40953 (2004).
22. Karastergiou, K.*, et al.* Distinct Developmental Signatures of Human Abdominal and Gluteal Subcutaneous Adipose Tissue Depots. *The Journal of Clinical Endocrinology & Metabolism* **98**, 362-371 (2013).
23. Schleinitz, D., Böttcher, Y., Blüher, M. & Kovacs, P. The genetics of fat distribution. *Diabetologia* **57**, 1276-1286 (2014).
24. Kumar, V., Sekar, M., Sarkar, P., Acharya, K.K. & Thirumurugan, K. Dynamics of HOX gene expression and regulation in adipocyte development. *Gene* **768**, 145308 (2021).
25. Tontonoz, P. & Spiegelman, B.M. Fat and beyond: the diverse biology of PPARgamma. *Annu Rev Biochem* **77**, 289-312 (2008).
26. Choy, L. & Derynck, R. Transforming growth factor-beta inhibits adipocyte differentiation by Smad3 interacting with CCAAT/enhancer-binding protein (C/EBP) and repressing C/EBP transactivation function. *J Biol Chem* **278**, 9609-9619 (2003).
27. Toyoda, S., Shin, J., Fukuhara, A., Otsuki, M. & Shimomura, I. Transforming growth factor beta1 signaling links extracellular matrix remodeling to intracellular lipogenesis upon physiological feeding events. *J Biol Chem* **298**, 101748 (2022).
28. He, Q.*, et al.* Regulation of HIF-1{alpha} activity in adipose tissue by obesity-associated factors: adipogenesis, insulin, and hypoxia. *American journal of physiology. Endocrinology and metabolism* **300**, E877-E885 (2011).
29. Geoghegan, G.*, et al.* Targeted deletion of Tcf7l2 in adipocytes promotes adipocyte hypertrophy and impaired glucose metabolism. *Mol Metab* **24**, 44-63 (2019).
30. Nguyen-Tu, M.S., Martinez-Sanchez, A., Leclerc, I., Rutter, G.A. & da Silva Xavier, G. Adipocyte-specific deletion of Tcf7l2 induces dysregulated lipid metabolism and impairs glucose tolerance in mice. *Diabetologia* **64**, 129-141 (2021).
31. Poulain-Godefroy, O.*, et al.* Inflammatory role of Toll-like receptors in human and murine adipose tissue. *Mediators Inflamm* **2010**, 823486 (2010).
32. Ferrari, F., Bock, P., Motta, M. & Helal, L. Biochemical and Molecular Mechanisms of Glucose Uptake Stimulated by Physical Exercise in Insulin Resistance State: Role of Inflammation. *Arquivos Brasileiros de Cardiologia* (2019).
33. Sethi, J.K. & Hotamisligil, G.S. The role of TNF alpha in adipocyte metabolism. *Semin Cell Dev Biol* **10**, 19-29 (1999).
34. Tukey, J.W., Ciminera, J.L. & Heyse, J.F. Testing the statistical certainty of a response to increasing doses of a drug. *Biometrics* **41**, 295-301 (1985).

GLASVEGAS ETHICS APPLICATION AND STUDY PROTOCOL

College of Medical, Veterinary & Life Sciences Ethics Committee for Non-Clinical Research Involving Human Subjects

APPLICATION FORM FOR ETHICAL APPROVAL

NOTES:
THIS APPLICATION FORM SHOULD BE TYPED NOT HAND WRITTEN.

ALL QUESTIONS MUST BE ANSWERED. "NOT APPLICABLE" IS A SATISFACTORY ANSWER WHERE APPROPRIATE.

Project Title:

The GlasVEGAs study: Glasgow Visceral & Ectopic fat with weight Gain in South Asians

Has this application been previously submitted to this or any other ethics committee?

No

Is this project from a commercial source, or funded by a research grant of any kind?

Yes project is fully funded by the European Commission as part of the European Medical Information Framework (EMIF).

If 'Yes', has it been referred to Research & Enterprise? Yes
Has it been allocated a project Number? Project number 60315/1
Give details, and ensure that this is stated on the Informed Consent Form.

Insurance Coverage and Restrictions:

The University insurance cover is restricted under specific circumstances, including the following -

- **work overseas.**
- **the use of hazardous materials.**
- **number of participants in excess of 5000.**

All such projects must be referred to Research & Enterprise and coverage confirmed before ethical approval is sought.

**Please tick here if this project has been referred to R&E to confirm adequate insurance coverage.**	N/A

Date of submission: 04/11/2014

Name of all person(s) submitting research proposal:

Dr James McLaren[1]
Dr Jason Gill[2]
Dr Dilys Freeman[3]
Dr John Foster[4]
Prof Naveed Sattar[5]

Position(s) held:

Clinical Research Fellow[1]
Reader in Exercise Science[2]
Senior Lecturer[3]
Honorary Lecturer/Deputy Head of MR Physics[4]
Professor of Metabolic Medicine[5]

School/Group/Institute/Centre:

[1,2,3,5] Institute of Cardiovascular and Medical Science
British Heart Foundation Glasgow Cardiovascular Research Centre

[4] School of Medicine/ NHS Greater Glasgow and Clyde

Address for correspondence relating to this submission:

Institute of Cardiovascular and Medical Science
British Heart Foundation Glasgow Cardiovascular Research Centre
Level 2, RM 224
126 University Place
Glasgow
G12 8TA

Email address: james.mclaren@glasgow.ac.uk

Name of Principal Researcher (if different from above, e.g., Student's Supervisor):

Prof Naveed Sattar

Position held: Professor of Metabolic Medicine

Undergraduate student project: No

Postgraduate student project: Yes - PhD Medicine

1. Describe the purposes of the research proposed. Please include the background and scientific justification for the research. Why is this an area of importance?

South Asians living in the United Kingdom have a 3-5 fold increased prevalence of type 2 diabetes, and develop the disease around a decade earlier and at a lower body mass index (BMI), compared to white Europeans [33,34]. In observational studies, the increase in diabetes risk per unit increase in BMI or waist circumference is substantially greater in South Asians than Europeans [35,36] suggesting that the adverse metabolic effects of increasing adiposity are greater in South Asians. Indeed, while South Asians carry more body fat than Europeans and this is often distributed more centrally [37-39] it appears that they remain more insulin resistant than Europeans after matching or adjustment for a range of adiposity markers [40,41] indicating that carrying the same amount of fat is associated with greater adverse metabolic consequences in South Asians than Europeans. However, these relatively crude adjustments for amount and location of adipose tissue do not account for potential differences in adipose tissue morphology or function that may influence cardio-metabolic disease risk. It has been hypothesised that South Asians have a lower capacity to store fat in the primary superficial subcutaneous adipose tissue compartment than Europeans and that this leads to earlier 'overflow' into secondary deep subcutaneous and visceral fat compartments [42], and potentially the liver. There is some evidence that South Asians may have greater amounts of deep subcutaneous abdominal adipose tissue than Europeans [42,43], however, this is not unequivocal [44]. Evidence that levels of visceral fat differ between South Asians and Europeans is also somewhat conflicting, with some studies reporting higher levels of visceral fat in South Asians [45,46], but other studies reporting no differences in visceral fat levels between the two ethnic groups [39,44,46]. Data are also conflicting concerning whether South Asians store a larger proportion of their abdominal fat in deep subcutaneous and visceral depots compared with Europeans, or whether their greater fat storage in deep subcutaneous and visceral depots simply reflects greater overall abdominal adiposity [43,44]. There have been some reports that South Asians may have higher levels of liver fat than Europeans [44,47], however, our recent data indicate that liver fat levels are not necessarily higher in South Asian than European men, despite the former group exhibiting greater insulin resistance (Ghouri et al, in preparation). Thus, overall the magnetic imaging spectroscopy (MRI) data are somewhat mixed and do not provide unequivocal support for earlier fat storage in secondary and ectopic compartments. However, the equivocal nature of these findings may reflect limitations in using single-slice MRI measurements for the accurate quantification of visceral fat [48-50] reducing statistical power to detect differences between the groups.

Observation of adipose tissue distribution does not provide information about underlying mechanisms responsible for the hypothesised reduced fat storage capacity in South Asians. Historically, two types of obesity have been described, termed hypertrophic obesity, in which the size of adipocytes increases, and hyperplastic obesity, in which adipocyte number increases. A body of evidence dating back to the 1970s has found that hypertrophic obesity is generally associated with increased insulin resistance and risk of diabetes, whereas hyperplastic obesity is more benign [48,51,52]. There is evidence that this may contribute to the insulin resistant phenotype in South Asians with Chandalia and colleagues reporting that South Asian men have a larger mean subcutaneous abdominal adipocyte size than Europeans [39], and Anand et al reporting that South Asians had greater subcutaneous abdominal adipocyte area and larger

adipocyte maximum diameter than Europeans [44]. However, recent evidence suggests that adipocyte size follows a bi-modal or tri-modal distribution, which is not adequately described by simply reporting mean adipocyte size [53-55]. These studies suggest that insulin resistant obesity is characterised by an increased proportion of small adipose cells [54,55] and larger large adipose cells [53,55]. This has been interpreted to reflect an inability for small adipose cells to terminally differentiate into mature adipose cells and increase triglyceride storage in insulin resistant individuals, which leads to increased size of the limited pool of large adipocytes and earlier storage in ectopic depots [54,55]. Indeed, there is evidence of reduced adipogenic gene expression in the insulin resistant obese [55,56]. Consistent with these reports, a recent study reported that South Asians were found to have both a higher ratio of small-to-larger adipocytes, and a larger fraction of very large adipocytes than Europeans [57]. Thus, it appears that a relative inability to increase triglyceride storage by increasing the population of fully differentiated large adipose cells may contribute to the insulin resistant phenotype in South Asians.

1. Describe the purposes of the research proposed. Please include the background and scientific justification for the research. Why is this an area of importance?

In response to an increase in fat mass, it appears that the initial response is hypertrophy of existing adipose cells, with an increase in cell number occurring in once there is limited capacity for further expansion [53,54,58]. Thus, in lean individuals, where there is capacity within existing adipose cells to grow, adipose cell hypertrophy is appropriate and can occur without major adverse consequences, but in obese individuals, recruitment *and* differentiation of precursor cells into mature and functional adipose cells is needed, to prevent overflow of lipid into secondary or ectopic depots. Thus, in the insulin resistant obesity phenotype, a reduced capacity for small adipose cells to increase triglyceride storage leads to an earlier accumulation of further small adipose cells. Recent data suggest that reduced upregulation lipid storage gene expression (e.g. DGAT2, CIDEA and SREBP1c) in subcutaneous adipose tissue and greater release of exogenous fatty acids into the circulation in response to overfeeding was associated with a larger increase visceral fat accumulation (27), providing evidence that an inability to increase lipid storage in subcutaneous adipose tissue leads to earlier deposition of fat into secondary depots during weight gain. We hypothesise that South Asians have an impaired capacity to generate expand small adipose cells and this leads to increased generation of small adipose cells at lower BMIs than occur in Europeans, with consequent effects on insulin resistance. Thus, we hypothesise, that while 'lean' Europeans (BMI < 25 $kg.m^{-2}$) will respond to modest short-term weight gain by increasing triglyceride storage in small adipose cells, thereby generating more mature adipose cells and 'safely' storing the excess fat, South Asians will increase the size of their existing large adipose cells and increase the number of small adipose cells, and have increased fat deposition in the visceral depot and the liver.

One limitation to the existing evidence on adiposity and insulin resistance in South Asians, is that the data are largely cross-sectional. One recent study reported that short-term (5 days) overfeeding with a high fat diet reduced insulin sensitivity in young lean South Asians (mean age: 22.2 years, mean BMI 20.9 $kg.m^{-2}$) but not in age and BMI-matched Europeans (28). No significant changes in subcutaneous or visceral fat were observed, and hepatic triglyceride content increased similarly in both groups, suggesting that this short-term effect was likely to be due to the acute effects of positive energy balance or high fat intake, rather than effects on adiposity. While these data suggest that South Asians have increased susceptibility to the adverse effects of a Westernised lifestyle, the effects of short-term positive energy balance are not necessarily analogous to weight gain, and thus the longer term effects of such an intervention, once weight gain has been induced, are unclear.

It is also unclear whether the physiological and metabolic changes associated with weight loss are similar for South Asians and Europeans. The higher gradient of the BMI vs diabetes risk relationship in South Asians (3;4), would suggest that South Asians may incur greater metabolic benefits from weight loss than Europeans, but this has not been tested in an intervention.

Therefore the aims of this study are to compare the physiological and metabolic changes associated with modest weight gain and weight loss in lean (BMI < 25 $kg.m^{-2}$) South Asian and European men. We hypothesise:

1) South Asian men will exhibit a greater adverse metabolic change (increased fasting glucose, insulin, TG, inflammatory markers and greater increases in glucose, insulin, TG and NEFA responses to a meal tolerance test) with modest weight gain than Europeans.
2) South Asians will have greater increases in visceral and liver fat deposition with modest weight gain than Europeans.
3) In response to weight gain Europeans will increase triglyceride storage in small adipose cells, thereby generating more mature adipose cells and ‘safely’ storing the excess fat, whereas South Asians will increase the size of their existing large adipose cells and increase the number of small adipose cells.
4) Changes in gene expression markers of adipogenesis in subcutaneous adipose tissue (e.g. WISP2, WNT, BMP4) and/or differences in regulation of lipid storage genes (e.g. DGAT2, CIDEA and SREBP1c) in subcutaneous adipose tissue of South Asians compared to Europeans in response to weight gain.
5) South Asians will exhibit greater positive metabolic changes with modest weight loss than Europeans.

2. Describe the design of the study and methods to be used. Include sample size and the calculation used to determine this. Statistical advice should be obtained if in doubt.

Participants will be men of European (n= 30) (self-report of both parents of white European origin) or South Asian (n = 30) (self-report of both parents of Indian, Pakistani, Bangladeshi or Sri Lankan origin), aged 18-45 years, with BMI <25 kg.m^2, who have been weight stable (± 2 kg) for >6 months (see power calculation below). Exclusion criteria will include diabetes (physician diagnosed or HbA1c ≥6.5% on screening), history of cardiovascular disease, regular participation in vigorous physical activity, current smoking, taking drugs or supplements thought to affect carbohydrate or lipid metabolism, or other significant illness that would prevent full participation in the study.

We will ask participants to gain 7% of baseline body weight over 4-6 weeks and then lose 7-15% of body weight over 12 weeks. We will assess body fat and fat distribution, adipose tissue function, metabolic responses to eating, energy expenditure, dietary intake, physical activity and fitness at three points during the study - at the start, after 7% weight gain and after 7-15% weight loss. Prior to each of the assessments the participants will be given a controlled 7 day weight neutral diet to ensure weight is stable. Exact energy requirements for the 7 day controlled diet will be calculated by doubly labelled water assessment. An overview of the study protocol is shown in Figure 1.

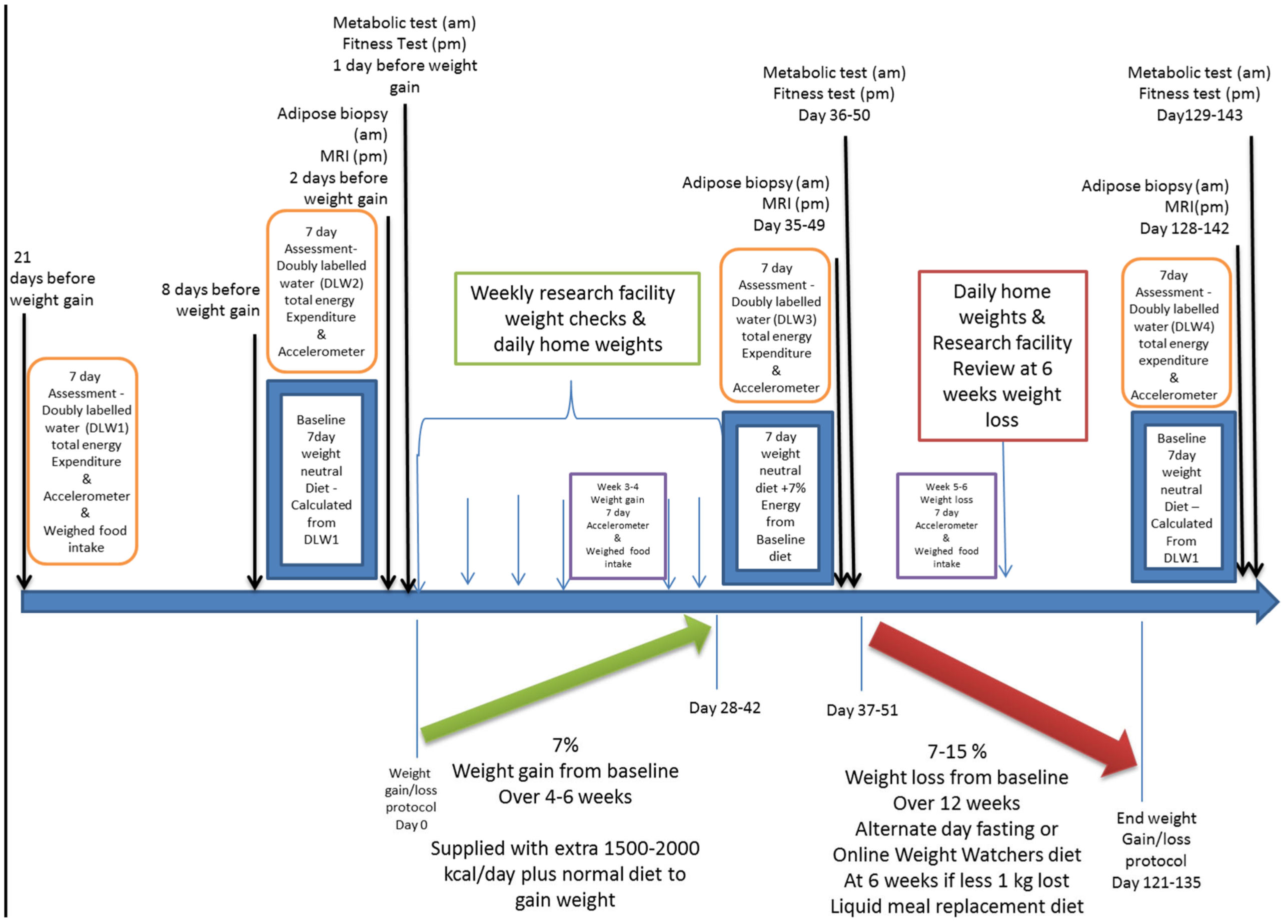

Figure 1. Overview of study protocol

2. Describe the design of the study and methods to be used. Include sample size and the calculation used to determine this. Statistical advice should be obtained if in doubt.

Assessments

1) **Measurement of energy expenditure using doubly labelled water (undertaken at 4 time-points; 3 weeks before baseline, baseline, after weight gain, after weight loss)**

Total energy expenditure of the participant will be assessed using doubly labelled water (DLW) over 7 days at four points during the study, as shown in Figure 1. Doubly labelled water is the gold-standard method to assess total energy expenditure and involves participants drinking water in which the oxygen is labelled with the oxygen-18 isotope and hydrogen is labelled with hydrogen-2 (deuterium). These are naturally occurring stable (i.e. non-radioactive) isotopes and consuming doubly-labelled water is completely safe. The water looks and tastes identical to normal water, but the heavier isotopes used mean that the label can be detected in body water (usually urine) samples and used to calculate energy expenditure [59]. Participants will be asked to drink a glass of doubly-labelled water and collect small urine samples every 1-2 days over the next 7 days. The enrichment of urine with the labelled hydrogen and oxygen will then be measured using mass spectrometry. As the labelled hydrogen is excreted entirely in body water, but the labelled oxygen is partially excreted in body water and partially in the form of carbon dioxide in response to oxidation of fuels for energy, the difference in the rates of excretion of the labelled hydrogen and oxygen in the urine can be used to calculate energy expenditure over the measurement period [60,61]. The measurement made 3 weeks before baseline will be used to determine food intake for the 7-day 'weight neutral diets' undertaken at baseline, after weight gain, and after weight loss.

2) **Weight neutral diets (undertaken at 3 time-points: baseline, after weight gain, after weight loss)**

During the 7-day DLW assessments made at baseline, after weight gain and after weight loss, participants will be provided with all of their food by experimenters, with the amount calculated to ensure a stable bodyweight during this period. For the baseline and post weight loss assessments, energy intake will equal the energy expenditure calculated from the DLW measurement made 3 weeks prior to baseline; for the post-weight gain assessments, the intake will equal 1.07 times this expenditure to take into account the increased energy requirements at the higher body weight. Diets provided will be tailored to take into account individual needs and preferences (religious, allergies, food likes/dislikes etc). During each 7-day period the participants will also wear an accelerometer to provide an objective measure of physical activity. Participants' body weight will be monitored daily throughout these 7-day periods and if weight changes by >0.5 kg over the week, energy intake may be adjusted and the monitoring period extended to ensure a 7-day weight stable period before metabolic assessment.

3) **Metabolic assessment and adipose tissue biopsy (undertaken at 3 time-points: baseline, after weight gain, after weight loss)**

At the end of each weight neutral diet period, participants will attend the metabolic investigation suite (Level 2 West Medical Building) for a metabolic assessment and adipose tissue biopsy. Participants will first have their metabolic rate and substrate utilisation assessed by indirect calorimetry using a ventilated hood, before a cannula will be inserted into an antecubital vein to enable a fasting blood sample to be taken.

Participants will then have an adipose tissue (fat) biopsy taken from subcutaneous adipose tissue in the abdomen. This involves numbing the skin with a local anaesthetic and then using a 'liposuction'

technique to remove ~1 g of fat (about the size of a baked bean) using a needle and syringe.[62] This is no more painful than having a blood sample taken and the biopsies will be performed by a medical doctor trained in this technique. The fat tissue will be analysed for adipocyte size distribution and number and expression of genes and proteins and metabolites involved with adipocyte formation, function, differentiation, growth, maturation, lipid processing and storage, metabolism and insulin sensitivity. Insulin sensitivity of the adipocytes at the cellular level will be calculated using an adipose tissue lipolysis assay previously described [63].

Participants will then be provided with a standard mixed test meal (containing ~800 kcal, 37% fat, 47% carbohydrate, 17% protein) and perform a mixed-meal tolerance test. Further blood samples will be taken for the next 5 hours to assess substrates and hormones related to glucose and lipid metabolism and biomarkers for cardio-metabolic disease risk (e.g. glucose, insulin, c-peptide, glucagon, lipid, adipokine, and NMR metabolomic responses).

4) **Body composition by magnetic resonance imaging (MRI) and magnetic imaging spectroscopy (MRS) (undertaken at 3 time-points: baseline, after weight gain, after weight loss)**

A whole-body multiple-slice MRI scan will be performed at baseline, after weight gain and after weight loss to quantify upper and lower body subcutenous adipose fat (including deep and superficial upper body subcutaneous fat) and visceral fat [64]. Liver fat and quadriceps intramuscular triglyceride will be quantified using ^{1}H MRS [64,65]. This will be undertaken using the MRI scanner in the BHF Glasgow Cardiovascular Research Centre, at the University of Glasgow.

5) **Fitness assessment (undertaken at 3 time-points: baseline, after weight gain, after weight loss)**

Participant will perform a continuous incremental uphill walking (or running, depending on fitness level) treadmill test to determine maximal oxygen uptake (VO_2max)[66].

Interventions

6) **Weight gain intervention**

After completion of baseline measurements, all participants will undergo an overfeeding protocol with the aim of inducing weight gain of ~7% body mass over ~4-6 weeks (~5kg in a 70 kg man). This will be achieved by asking participants to eat until they felt more full than usual and providing them with high energy snacks (premium ice cream, chocolate bars, potato crisps, cheese, dried fruit and nuts, sugary drinks) to supplement their usual food intake by 1500-2000 kcal/day. During this time, participant's weight will be recorded daily at home, and weekly in the lab, to assess rate of rate gain. During these weekly visits snacks for the following week will be provided. At week 3-4 of weight gain, participants will be asked to perform a 7-day weighed food record to assess energy intake and wear an accelerometer to assess physical activity. Once participants have gained 7% bodyweight, they will be place on a weight neutral diet for 7 days (see point 2 above) before the post weight gain assessments are made,

7) **Weight loss intervention**

After completion of the post weight gain measurements, all participants will undergo a weight loss intervention, with the aim of inducing 7-15% weight loss from peak weight. During this time, participant's weight will be recorded daily at home to monitor progress. As it has been shown that most dietary interventions induce similar weight loss provided that they are adhered to [67], participants will be provided an option of two dietary programmes, either an online Weight Watchers diet (www.weightwatchers.co.uk) or an alternative day fasting diet [68], based on personal preference. Both diets have been shown to be effective in inducing mean weight loss of >5kg in over this time frame [67,68]. The weight watchers diet uses a points system whereby a portion of each food is assigned points score and participants are provided with a personalised overall target number of daily points that they should aim to stay below. The alternative day fasting diet is based on the protocol described by Varady et al [68], in which participants reduce energy intake to ~500 kcal/per day (~25% of energy requirements) on days and on non-fasting days participants will be allowed to eat ad libitum. Participants will be advised to try and avoid staying up late or getting up early specifically to eat extra food prior to or after fasting days. To accompany the dietary intervention, participants will be asked to increase physical activity to expend an extra ~1800-2500 kcal/week, in line with national guidance [69]. This will be facilitated by providing participants with a pedometer and asking them to increase baseline physical activity by 4000-6000 steps per day (~40-60 minutes of walking) on 5 days of the week over the course of the intervention. At week 5-6 of weight loss, participants will be asked to perform a 7-day weighed food record to assess energy intake and wear an accelerometer to assess physical activity. After 6 weeks of dietary intervention

if the participants have not lost at least 1 kg they will be offered to change to a complete meal replacement diet to ensure they achieve 7-15% of weight loss at 12 weeks. If the participants are still not at baseline weight by the end of protocol we will offer them ongoing dietary and exercise support after the study.

Power calculation
Twenty three participants per group will enable detection of a 1 SD difference in change in an outcome measure with weight gain or weight loss between groups with 90% power at $p < 0.05$ (n = 17 for 80% power). To put this effect size into perspective, the decrease in insulin sensitivity in response to 5 days of overfeeding was ~20-fold greater (20% vs 1% change) in South Asians than Europeans, a difference which was significant at the $p < 0.005$ level with groups of 12 participants (SD for the change not given in the paper)[70]. Furthermore, cross-sectionally the differences in number of small adipocytes and in the ratio of small-to-large adipocytes between South Asians and Europeans are ~1.5-2 SDs of the population mean values [57]. To allow for 30% dropout over the course of the intervention, we propose to recruit 30 participants per group.

3. Describe the research procedures as they affect the research subject and any other parties involved. It should be clear exactly (i) what will happen to the research participant, (ii) how many times and (iii) in what order.

Screening visit (Visit 1) – Explanation of study including screening with inclusion/exclusion criteria. Given volunteer information sheet and written informed consent obtained.
Visit 2 – Start initial doubly labelled water/energy assessment/accelerometer/weighed food diary (DLW1) for 7 days eating normal diet.
Visit 3 – End of 7 day doubly labelled water 1 assessment, hand in food diary and urine samples for DLW1.
Visit 4 – Start of baseline weight neutral diet for 7 days – given 7 day supply of food. Repeat 7 day doubly labelled water assessment /accelerometer/ weighed food diary (DLW2).
Visits 5 & 6 – End of baseline weight neutral diet & hand in food diary & urine samples for DLW2. Participants attend consecutive days for metabolic / fitness tests and MRI/adipose biopsy. Start of weight gain protocol.
Visits 7 - 9/11 – Weight gain protocol lasts 4-6 weeks, participants attend weekly for research facility review/weight and participants monitor daily weights at home. Week 3 to 4 undertake 7 day weighed food diary and accelerometer.
Visit 10/12- End weight gain protocol. Given food for baseline 7 day weight neutral diet +7% energy and doubly labelled water/energy assessment / accelerometer /weighed food diary (DLW3).
Visits 11/13 & 12/14 - End of baseline weight neutral diet +7% energy & hand in food diary & urine samples for DLW3. Participants attend consecutive days for metabolic / fitness tests and MRI/adipose biopsy. Start of weight loss protocol.
Visit 13/15 – Weight loss protocol lasts 12 weeks, participants attend after 6 weeks. Week 5 to 6 undertake 7 day weighed food diary and accelerometer.
Visit 14/16 – End weight loss protocol after 12 weeks. Given food for baseline 7 day weight neutral diet and doubly labelled water /energy assessment /accelerometer /weighed food diary (DLW4).
Visits 15/17 & 16/18 - End of baseline weight neutral diet & hand in food diary & urine samples for DLW4. Participants attend consecutive days for metabolic / fitness tests and MRI/adipose biopsy. End of study.

4. How will potential participants in the study be (i) identified, (ii) approached and (iii) recruited? Give details for cases and controls separately if appropriate.

Participants will be recruited via personal contact and local advertising. Advertisements will be placed in the University of Glasgow website and local newsletter and sent to staff e-mail lists. Flyers will be placed around the University campus and in locations throughout the city. If recruitment is slow, we may consider advertising in a local newspaper. Participants will initially be contacted by phone and will be screened at visit 1 according to inclusion and exclusion criteria.

Participants will be men of European (self-report of both parents of white European origin) or South Asian (self-report of both parents of Indian, Pakistani, Bangladeshi or Sri Lankan origin), aged 18-45 years, with BMI <25 $kg.m^2$, who have been weight stable (± 2 kg) for >6 months. Exclusion criteria will include diabetes (physician diagnosed or HbA1c ≥6.5% on screening), history of cardiovascular disease, regular participation in vigorous physical activity, current smoking, taking drugs or supplements thought to affect carbohydrate or lipid metabolism, or other significant illness that would prevent full participation in the study.

Participants will be fully informed about the risks, burdens and benefits of the study explained by study doctor James McLaren or another of the study investigators (Naveed Sattar, Jason Gill). They will be given a patient information sheet about all aspects of the study, be shown the 14 gauge needle that will be used for adipose tissue biopsy, and have the opportunity to ask questions before providing written informed consent to take part in the study.

5. What are the ethical considerations involved in this proposal? You may wish, for example, to comment on issues to do with consent, confidentiality, risk to subjects, etc.

Weight gain During the study we will ask participants to gain 7% of their initial body weight over ~6 weeks (~5 kg in a 70 kg man). This experimental approach has been adopted by number of studies previously [58,71-77]. Weight gain is likely to temporarily induce adverse metabolic changes (e.g. increase insulin resistance and blood lipids). However, this weight gain will only be temporary and we will support participants in losing this weight over the subsequent 12 weeks, aiming to have them return to a weight lower than their baseline weight by the end of the study. Participants will be given the choice of undertaking one of two weight loss programmes (weight watchers or alternate-day fasting), which have been shown to be clinically effective in inducing weight loss of this magnitude over this time period [67,68]. Participants will be weighed throughout this weight loss phase, and any person losing < 1 kg within the first 6 weeks of the weight loss period will be given the choice to move to a total meal replacement diet to facilitate more rapid weight loss.[78] Any participant who does not reach their baseline weight by the end of the 12-week weight loss phase, we will support them to continue with their choice of a Weight Watchers, alternate day fasting or complete meal replacement diet, for a further 12 weeks to help them to return to return to baseline weight. There is no evidence that short term temporary weight gain induces any long-term adverse effects but to further minimise the potential for any long-term effects of temporary weight gain, we will only include young (age 18-45 years), normal bodyweight individuals (BMI < 25 $kg.m^{-2}$), without diabetes, cardiovascular disease or other significant medical illness at baseline. In addition, the participants will gain the knowledge during the weight loss phase of the study of approaches that they may be able to use to help control their weight in the future. If a complication such as hypertension, diabetes or cardiovascular disease does develop then initial assessment will take place in the BHF clinical research facility and onwards referral to the participant's general practitioner for further assessment and ongoing management in NHS primary or secondary care will take place.

Adipose biopsies - We will be taking three adipose biopsies of abdominal subcutaneous fat from participants during the study. This is performed using a liposuction technique. The skin is anaesthetised with 2% lignocaine and an adipose tissue sample (~1 g) is aspirated through a 14-gauge needle attached to a 50 ml syringe. This is a relatively painless procedure. There is a small risk of local infection and minor bruising but this is minimised by aseptic (sterile) practice and application of ice and pressure to the site for 5-10 post-biopsy. Adipose biopsies will be performed by Dr James McLaren MRCP, a medical doctor who has been trained in this technique (or another appropriately trained medically qualified person). The investigators have extensive experience in carrying out these biopsies (>200 biopsies performed) successfully without adverse consequence in previous studies. Adipose tissue biopsies may be somewhat more invasive than procedures usually undertaken in studies reviewed by the MVLS ethics committee. We have liaised with the NHS ethics committee and Prof Billy Martin (Chair of the MVLS ethics committee) about which committee this application should be sent to, and the agreement was that the MVLS ethics committee was most appropriate. Please see the email correspondence on this in the appendix at the end of this form.

Venous cannulation This incurs a minor risk of bruising. There is also a risk of thrombophlebitis (inflammation of the vein) but this risk is small in non-smokers with no history of coagulation disorders. Plastic or air embolism can occur if incorrect cannulation technique is employed but good practice minimizes this risk. Some individuals may feel faint when giving blood.

Exercise tests - One part of the exercise test will be at a maximal level. Given the relatively young age of the subjects (18–45 years) maximal exercise testing is low risk in this subject group. Preliminary screening will exclude any subjects with a history of cardiovascular problems and those known to exhibit major risk factors for coronary heart disease. Heart rate will be continuously monitored during the tests which will take place in a laboratory containing a defibrillator, emergency drugs and a telephone to contact emergency services, in the unlikely event of a problem arising.

5. What are the ethical considerations involved in this proposal? You may wish, for example, to comment on issues to do with consent, confidentiality, risk to subjects, etc.

Magnetic resonance imaging/spectroscopy - Magnetic resonance imaging is not harmful to the participants and involves lying flat in the magnetic resonance image (MRI) scanner for 45 minutes. There can be a sensation of body warmth when the scanner is on and the scanner can be noisy although headphones are provided to listen to the radio or music during the scan. There is no radiation involved and the images are obtained by a large magnet which excites hydrogen atoms in the body without damaging or altering the structure of body cells. The excited hydrogen atoms then give off a radio-frequency signal which varies between different body parts and is used to create an image of the body tissues and organs. Because a magnet is used to create the images contraindications to MRI include metallic implants or shrapnel in the body, cochlear implants or a cardiac pacemaker.

Doubly labelled water – Doubly labelled water is water which has the chemical properties of the elements of hydrogen and oxygen slightly altered so they can be measured – called an isotope. This isotope is not radioactive and the water is entirely safe. The oxygen isotope is an oxygen-18 isotope and the hydrogen is an isotope with the element deuterium. Water with similar chemical properties occurs in nature and is not harmful in any way.

6. Outline the reasons why the possible benefits to be gained from the project justify any risks or discomforts involved.

Diabetes is a major public health problem, particularly in South Asians, so increasing understanding of the mechanisms linking weight gain to adverse metabolic consequences is of major public health importance. Participants in the study will receive feedback with detailed health, dietary and fitness information and will be guided through a weight loss programme that will equip them with tools to help them to maintain a heathy weight in the future. Thus we feel that the benefits of the study greatly outweigh the risks from both a scientific/public health perspective and the perspective of health benefits to the individual participant.

7. Who are the investigators (including assistants) who will conduct the research? What are their qualifications and experience?

Dr James McLaren, MBChB, MRCP registrar in endocrinology & diabetes has 10 years' experience in venepuncture and cannulation, general medicine and the hospital specialty of endocrinology & diabetes medicine. The research group undertaking the research has performed greater than 200 adipose tissue biopsies in recent studies over the last 10 years and Dr James McLaren will be taught and initially supervised to ensure full competence in the procedure.

Prof Naveed Sattar MBChB, MRCPath, FRCP, PhD is Professor of metabolic medicine and has extensive experience in conducting human metabolic studies, cardiovascular disease biomarker and diabetes related research over the past 20 years.

Dr Jason Gill, BSc (Hons), MSc, PhD has ~17 years of experience in conducting human metabolic and exercise studies and has ~14 years experience in venepuncture and cannulation.

Dr Dilys Freeman BSc, PhD has expertise in the area of adipose tissue analysis and processing and will be working in collaboration with Prof Ulf Smith at the University of Gothenburg.

Dr John Foster PhD, CSci, MIPEM has extensive experience in magnetic resonance imaging research and clinical studies. Will be advising and supervising use of MRI and MRS for assessment of fat analysis.

<u>International collaborators:</u>

Prof Ulf Smith (University of Gothenburg, Sweden) and his team at the Lundberg laboratory for Diabetes research will be analysing adipose tissue biopsy samples for signalling pathways relevant to adipocyte function, formation and differentiation.

Prof Ele Ferraninni (University of Pisa, Italy) and his team at the Metabolism Unit at the Institute of Clinical Physiology, Italy will be providing expertise and analysis in doubly labelled water/energy assessment and mixed meal/ metabolic assessment of participants.

8. Are arrangements for the provision of clinical facilities to handle emergencies necessary? If so, briefly describe the arrangements made.

The clinical research facility contains a defibrillator, emergency drugs and a telephone to contact emergency services, in the unlikely event of a problem arising. The fitness test will be carried out in a laboratory in the clinical research facility with the participants heart rate monitored at all times.

9. In cases where subjects will be identified from information held by another party (e.g., a doctor or hospital), describe how you intend to obtain this information. Include, where appropriate, which Multi Centre Research Ethics Committee or Local Research Ethics Committee will be applied to.

Not applicable to this study.

10. Specify whether subjects will include students or others in a dependent relationship and, where possible, avoid recruiting students who might feel to be, or be construed to be, under obligation to volunteer for a project. This is most likely to be when a student is enrolled on a course where the investigator is a teacher. In these circumstances, the recruitment could be carried out by one of the other investigators or a suitably qualified third party.

If students are enrolled on a course or supervised by one of the study investigators, recruitment will be carried out by another member of the study team who is not directly involved with their studies and therefore the participants will be under no obligation to volunteer for the study.

11. Specify whether the research will include children or participants with mental illness, disability or handicap. If so, please explain the necessity of involving these individuals as research subjects and include documentation of the suitability of those researchers who will be in contact with children (e.g., Disclosure Scotland or membership of the PVG Scheme).

The research/study will not include children or participants with mental illness, disability or handicap.

12. Will payment or other incentive, such as a gift or free services, be made to any research subject? If so, please specify, and state the level of payment to be made and/or the source of the funds/gift/free service to be used. Please explain the justification for offering an incentive.

As this study requires a large commitment from each individual subject, we will provide participants with a contribution of £200 after the weight gain phase of the study and a further £200 after the weight loss phase of the study as a thank you for the inconvenience that participation in the study would have caused them.

13. Please give details of how consent is to be obtained. A copy of the proposed consent form, along with a separate information sheet, written in simple, non-technical language MUST ACCOMPANY THIS PROPOSAL FORM.

Dr James McLaren (main study doctor) or one of the study investigators (Prof Naveed Sattar or Dr Jason Gill) will seek informed written consent. This will involve participants receiving a written volunteer information sheet with information about all aspects of the study. Full benefits, burdens and risks of the study will be explained prior to obtaining written consent on the volunteer consent form.

14. Comment on any cultural, social or gender-based characteristics of the subjects which have affected the design of the project or may affect its conduct.

Participants will be recruited on the basis of their ethnic origin (South Asian or white European) as this reflects the main hypothesis of the study.

Dietary advice and the snack options for the weight gain protocol will be broad including different options to account for the different typical South Asian and European diets.

15. Please state (i) who will have access to the data, (ii) how the data will be stored, how will access be restricted and how long will it be retained, and (iii) what measures will be adopted to maintain the confidentiality of the research subjects and to comply with data protection requirements.

i) Only the named investigators will have full access to the data obtained. The information obtained will be anonymised and identifiable data will not be passed on to anyone outside the study group. Anonymised data, only coded with a participant information number, with all identifying information removed, will be shared with collaborators to maximise the life-time value of the data generated for human health, in line with best practice for data sharing.

ii) Data will be stored on personal computers of the named investigators on the University server. Access will be restricted by password-protection and data will be stored for 10 years.

Iii) Data will be anonymised with subjects being identified by a number rather than a name. Personal data including linkage of subject name and number will be held in a locked filing cabinet in the BHF Glasgow clinical research centre or as password−protected files saved on the University server, known only to the named investigators.

In regard to (ii) above, please clarify (tick one) how the data will be stored:

☐ **(a) in a fully anonymised form (link to subject broken),**

☐ **(b) in a linked anonymised form (data +/- samples linked to subject identification number but subject not identifiable to researchers), or**

X (c) in a form in which the subject could be identifiable to researcher.

If data are stored in linked anonymised form, please state who will have access to the code and personal information about the subject.

16. To your knowledge, will the intended group of research subjects be involved in other research? If so, please justify.

No

17. Proposed starting date: 1 December 2014

Expected completion date: 30 November 2017

18. Please state location(s) where the project will be carried out.

West Medical Building and British Heart Foundation Glasgow Cardiovascular Research Centre at the University of Glasgow.

19. Please state briefly any precautions being taken to protect the health and safety of researchers and others associated with the project (as distinct from the research subjects), e.g., where blood samples are being taken.

There is a risk of transmission of blood−borne infection to the researcher when blood samples are being taken. However, this is minimised by good laboratory practice and following local health and safety regulations.

20. Please state all relevant sources of funding or support for this study.

Project is funded by a grant from the EMIF (European Medical Information Framework).

21a). Are there any conflicts of interest related to this project for any member of the research team? This includes, but is not restricted to, financial or commercial interests in the findings. If so, please explain these in detail and justify the role of the research team. For each member of the research team please complete a declaration of conflicts of interest below.

Researcher Name: Dr James Mclaren - conflict of interest - None

Researcher Name: Dr Naveed Sattar - conflict of interest - None

Researcher Name: Dr Jason Gill - conflict of interest - None

Researcher Name: Dr Dilys Freeman - conflict of interest - None

Researcher Name: Dr John Foster - conflict of interest - None

21b). If there are any conflicts of interest, please describe these in detail and justify conducting the proposed study.

No conflict of interests to declare.

22. How do you intend to disseminate the findings of this research?

The work and results of the research will be disseminated by publications in peer-reviewed scientific journals, internal report and presentations at conferences. The research participants will also receive written feedback and the research will form the basis for the PhD thesis by Dr James McLaren.

I confirm that have read the University of Glasgow's Data Protection Policy.
[http://www.gla.ac.uk/services/dpfoioffice/policiesandprocedures/dpa-policy/]

Please initial box JM

Name James MacLaren Date 04.11.14
(Proposer of research)
Please type your name on the line above.

For student projects:

I confirm that I have read and contributed to this submission and believe that the methods proposed and ethical issues discussed are appropriate.

I confirm that the student will have the time and resources to complete this project.

Name ____ Date __________________
(Supervisor of student)
Please type your name on the line above.

Please upload the completed and <u>signed</u> form, along with other required documents by logging in to the Research Ethics System at - https://frontdoor.spa.gla.ac.uk/login/

References:

1. Diez, J.J. & Iglesias, P. The role of the novel adipocyte-derived hormone adiponectin in human disease. *European Journal of Endocrinology Eur J Endocrinol* **148**, 293-300 (2003).
2. Huang, Z.H., Reardon, C.A., Getz, G.S., Maeda, N. & Mazzone, T. Selective suppression of adipose tissue apoE expression impacts systemic metabolic phenotype and adipose tissue inflammation. *J Lipid Res* **56**, 215-226 (2015).
3. Huang, Z.H., Reardon, C.A. & Mazzone, T. Endogenous ApoE expression modulates adipocyte triglyceride content and turnover. *Diabetes* **55**, 3394-3402 (2006).
4. Zhao, H.*, et al.* A novel promoter controls Cyp19a1 gene expression in mouse adipose tissue. *Reprod Biol Endocrinol* **7**, 37 (2009).
5. Ding, Q., Gupta Rajat, M., Raghavan, A. & Musunuru, K. Abstract 70: KLF14 is a Novel Regulator of Human Metabolism. *Arteriosclerosis, Thrombosis, and Vascular Biology* **34**, A70-A70 (2014).
6. Hofmann, S.M.*, et al.* Adipocyte LDL receptor-related protein-1 expression modulates postprandial lipid transport and glucose homeostasis in mice. *J Clin Invest* **117**, 3271-3282 (2007).
7. Harris, R.B. Direct and indirect effects of leptin on adipocyte metabolism. *Biochim Biophys Acta* **1842**, 414-423 (2014).
8. Gonzales, A.M. & Orlando, R.A. Role of adipocyte-derived lipoprotein lipase in adipocyte hypertrophy. *Nutr Metab (Lond)* **4**, 22 (2007).
9. Stienstra, R.*, et al.* The inflammasome-mediated caspase-1 activation controls adipocyte differentiation and insulin sensitivity. *Cell Metab* **12**, 593-605 (2010).
10. Abreu-Vieira, G.*, et al.* Cidea improves the metabolic profile through expansion of adipose tissue. *Nat Commun* **6**, 7433 (2015).
11. Zhou, Z.*, et al.* Estrogen receptor α controls metabolism in white and brown adipocytes by regulating Polg1 and mitochondrial remodeling. *Science Translational Medicine* **12**, eaax8096 (2020).
12. Vieira-Potter, V.J., Zidon, T.M. & Padilla, J. Exercise and Estrogen Make Fat Cells "Fit". *Exerc Sport Sci Rev* **43**, 172-178 (2015).
13. Glad, C.A.M.*, et al.* Expression of GHR and Downstream Signaling Genes in Human Adipose Tissue-Relation to Obesity and Weight Change. *J Clin Endocrinol Metab* **104**, 1459-1470 (2019).
14. Ridderstrale, M. Signaling Mechanism for the Insulin-like Effects of Growth Hormone - Another Example of a Classical Hormonal Negative Feedback Loop. *Current drug targets. Immune, endocrine and metabolic disorders* **5**, 79-92 (2005).
15. Li, P., Wang, Y., Zhang, L., Ning, Y. & Zan, L. The Expression Pattern of PLIN2 in Differentiated Adipocytes from Qinchuan Cattle Analysis of Its Protein Structure and Interaction with CGI-58. *Int J Mol Sci* **19**(2018).
16. Hui, X.*, et al.* Adipocyte SIRT1 controls systemic insulin sensitivity by modulating macrophages in adipose tissue. *EMBO Rep* **18**, 645-657 (2017).
17. Salminen, A., Kaarniranta, K. & Kauppinen, A. Crosstalk between Oxidative Stress and SIRT1: Impact on the Aging Process. *Int J Mol Sci* **14**, 3834-3859 (2013).
18. Payne, V.A.*, et al.* The human lipodystrophy gene BSCL2/seipin may be essential for normal adipocyte differentiation. *Diabetes* **57**, 2055-2060 (2008).

19. Shimba, S., Wada, T., Hara, S. & Tezuka, M. EPAS1 promotes adipose differentiation in 3T3-L1 cells. *J Biol Chem* **279**, 40946-40953 (2004).
20. Karastergiou, K.*, et al.* Distinct Developmental Signatures of Human Abdominal and Gluteal Subcutaneous Adipose Tissue Depots. *The Journal of Clinical Endocrinology & Metabolism* **98**, 362-371 (2013).
21. Schleinitz, D., Böttcher, Y., Blüher, M. & Kovacs, P. The genetics of fat distribution. *Diabetologia* **57**, 1276-1286 (2014).
22. Kumar, V., Sekar, M., Sarkar, P., Acharya, K.K. & Thirumurugan, K. Dynamics of HOX gene expression and regulation in adipocyte development. *Gene* **768**, 145308 (2021).
23. Tontonoz, P. & Spiegelman, B.M. Fat and beyond: the diverse biology of PPARgamma. *Annu Rev Biochem* **77**, 289-312 (2008).
24. Choy, L. & Derynck, R. Transforming growth factor-beta inhibits adipocyte differentiation by Smad3 interacting with CCAAT/enhancer-binding protein (C/EBP) and repressing C/EBP transactivation function. *J Biol Chem* **278**, 9609-9619 (2003).
25. Toyoda, S., Shin, J., Fukuhara, A., Otsuki, M. & Shimomura, I. Transforming growth factor beta1 signaling links extracellular matrix remodeling to intracellular lipogenesis upon physiological feeding events. *J Biol Chem* **298**, 101748 (2022).
26. He, Q.*, et al.* Regulation of HIF-1{alpha} activity in adipose tissue by obesity-associated factors: adipogenesis, insulin, and hypoxia. *American journal of physiology. Endocrinology and metabolism* **300**, E877-E885 (2011).
27. Geoghegan, G.*, et al.* Targeted deletion of Tcf7l2 in adipocytes promotes adipocyte hypertrophy and impaired glucose metabolism. *Mol Metab* **24**, 44-63 (2019).
28. Nguyen-Tu, M.S., Martinez-Sanchez, A., Leclerc, I., Rutter, G.A. & da Silva Xavier, G. Adipocyte-specific deletion of Tcf7l2 induces dysregulated lipid metabolism and impairs glucose tolerance in mice. *Diabetologia* **64**, 129-141 (2021).
29. Poulain-Godefroy, O.*, et al.* Inflammatory role of Toll-like receptors in human and murine adipose tissue. *Mediators Inflamm* **2010**, 823486 (2010).
30. Ferrari, F., Bock, P., Motta, M. & Helal, L. Biochemical and Molecular Mechanisms of Glucose Uptake Stimulated by Physical Exercise in Insulin Resistance State: Role of Inflammation. *Arquivos Brasileiros de Cardiologia* (2019).
31. Sethi, J.K. & Hotamisligil, G.S. The role of TNF alpha in adipocyte metabolism. *Semin Cell Dev Biol* **10**, 19-29 (1999).
32. Tukey, J.W., Ciminera, J.L. & Heyse, J.F. Testing the statistical certainty of a response to increasing doses of a drug. *Biometrics* **41**, 295-301 (1985).
33. Mukhopadhyay, B., Forouhi, N.G., Fisher, B.M., Kesson, C.M. & Sattar, N. A comparison of glycaemic and metabolic control over time among South Asian and European patients with Type 2 diabetes: results from follow-up in a routine diabetes clinic. *Diabetic medicine : a journal of the British Diabetic Association* **23**, 94-98 (2006).
34. Sproston, K.a.M., J. The health of minority ethnic groups. in *Health Survey for England 2004*, Vol. 1 (2006).
35. Ntuk, U.E., Gill, J.M., Mackay, D.F., Sattar, N. & Pell, J.P. Ethnic-Specific Obesity Cutoffs for Diabetes Risk: Cross-sectional Study of 490,288 UK Biobank Participants. *Diabetes care* (2014).
36. Chiu, M., Austin, P.C., Manuel, D.G., Shah, B.R. & Tu, J.V. Deriving ethnic-specific BMI cutoff points for assessing diabetes risk. *Diabetes care* **34**, 1741-1748 (2011).

37. McKeigue, P.M., Shah, B. & Marmot, M.G. Relation of central obesity and insulin resistance with high diabetes prevalence and cardiovascular risk in South Asians. *Lancet* **337**, 382-386 (1991).
38. Misra, A. & Vikram, N.K. Insulin resistance syndrome (metabolic syndrome) and obesity in Asian Indians: evidence and implications. *Nutrition* **20**, 482-491 (2004).
39. Chandalia, M.*, et al.* Insulin resistance and body fat distribution in South Asian men compared to Caucasian men. *PloS one* **2**, e812 (2007).
40. Hall, L.M.*, et al.* Fat oxidation, fitness and skeletal muscle expression of oxidative/lipid metabolism genes in South Asians: implications for insulin resistance? *PloS one* **5**, e14197 (2010).
41. Forouhi, N.G.*, et al.* Relation of triglyceride stores in skeletal muscle cells to central obesity and insulin sensitivity in European and South Asian men. *Diabetologia* **42**, 932-935 (1999).
42. Sniderman, A.D., Bhopal, R., Prabhakaran, D., Sarrafzadegan, N. & Tchernof, A. Why might South Asians be so susceptible to central obesity and its atherogenic consequences? The adipose tissue overflow hypothesis. *International journal of epidemiology* **36**, 220-225 (2007).
43. Kohli, S., Sniderman, A.D., Tchernof, A. & Lear, S.A. Ethnic-specific differences in abdominal subcutaneous adipose tissue compartments. *Obesity* **18**, 2177-2183 (2010).
44. Anand, S.S.*, et al.* Adipocyte hypertrophy, fatty liver and metabolic risk factors in South Asians: the Molecular Study of Health and Risk in Ethnic Groups (mol-SHARE). *PloS one* **6**, e22112 (2011).
45. Lear, S.A., Chockalingam, A., Kohli, S., Richardson, C.G. & Humphries, K.H. Elevation in cardiovascular disease risk in South Asians is mediated by differences in visceral adipose tissue. *Obesity* **20**, 1293-1300 (2012).
46. Forouhi, N.G., Sattar, N. & McKeigue, P.M. Relation of C-reactive protein to body fat distribution and features of the metabolic syndrome in Europeans and South Asians. *International journal of obesity and related metabolic disorders : journal of the International Association for the Study of Obesity* **25**, 1327-1331 (2001).
47. Petersen, K.F.*, et al.* Increased prevalence of insulin resistance and nonalcoholic fatty liver disease in Asian-Indian men. *Proceedings of the National Academy of Sciences of the United States of America* **103**, 18273-18277 (2006).
48. So, R.*, et al.* Multiple-slice magnetic resonance imaging can detect visceral adipose tissue reduction more accurately than single-slice imaging. *European journal of clinical nutrition* **66**, 1351-1355 (2012).
49. Duffy, A., Schurr, M., Warner, T. & Chen, H. Long-term outcomes in patients with calciphylaxis from hyperparathyroidism. *Annals of surgical oncology* **13**, 96-102 (2006).
50. Greenfield, J.R., Samaras, K., Chisholm, D.J. & Campbell, L.V. Regional intra-subject variability in abdominal adiposity limits usefulness of computed tomography. *Obesity research* **10**, 260-265 (2002).
51. Arner, P., Arner, E., Hammarstedt, A. & Smith, U. Genetic predisposition for Type 2 diabetes, but not for overweight/obesity, is associated with a restricted adipogenesis. *PloS one* **6**, e18284 (2011).
52. Lundgren, M.*, et al.* Fat cell enlargement is an independent marker of insulin resistance and 'hyperleptinaemia'. *Diabetologia* **50**, 625-633 (2007).

53. Yang, J., Eliasson, B., Smith, U., Cushman, S.W. & Sherman, A.S. The size of large adipose cells is a predictor of insulin resistance in first-degree relatives of type 2 diabetic patients. *Obesity* **20**, 932-938 (2012).
54. McLaughlin, T.*, et al.* Enhanced proportion of small adipose cells in insulin-resistant vs insulin-sensitive obese individuals implicates impaired adipogenesis. *Diabetologia* **50**, 1707-1715 (2007).
55. McLaughlin, T.*, et al.* Subcutaneous adipose cell size and distribution: relationship to insulin resistance and body fat. *Obesity* **22**, 673-680 (2014).
56. Gustafson, B., Hammarstedt, A., Hedjazifar, S. & Smith, U. Restricted adipogenesis in hypertrophic obesity: the role of WISP2, WNT, and BMP4. *Diabetes* **62**, 2997-3004 (2013).
57. Balakrishnan, P., Grundy, S.M., Islam, A., Dunn, F. & Vega, G.L. Influence of upper and lower body adipose tissue on insulin sensitivity in South Asian men. *Journal of investigative medicine : the official publication of the American Federation for Clinical Research* **60**, 999-1004 (2012).
58. Tchoukalova, Y.D.*, et al.* Regional differences in cellular mechanisms of adipose tissue gain with overfeeding. *Proceedings of the National Academy of Sciences of the United States of America* **107**, 18226-18231 (2010).
59. Schoeller, D.A. & van Santen, E. Measurement of energy expenditure in humans by doubly labeled water method. *Journal of applied physiology: respiratory, environmental and exercise physiology* **53**, 955-959 (1982).
60. Levine, J.A. Measurement of energy expenditure. *Public health nutrition* **8**, 1123-1132 (2005).
61. Ritz, P. & Coward, W.A. Doubly labelled water measurement of total energy expenditure. *Diabete & metabolisme* **21**, 241-251 (1995).
62. Moran, C.N.*, et al.* Effects of diabetes family history and exercise training on the expression of adiponectin and leptin and their receptors. *Metabolism: clinical and experimental* **60**, 206-214 (2011).
63. Huda, S.S.*, et al.* In preeclampsia, maternal third trimester subcutaneous adipocyte lipolysis is more resistant to suppression by insulin than in healthy pregnancy. *Hypertension* **63**, 1094-1101 (2014).
64. Thomas, E.L.*, et al.* Hepatic triglyceride content and its relation to body adiposity: a magnetic resonance imaging and proton magnetic resonance spectroscopy study. *Gut* **54**, 122-127 (2005).
65. Roldan-Valadez, E.*, et al.* In vivo 3T spectroscopic quantification of liver fat content in nonalcoholic fatty liver disease: Correlation with biochemical method and morphometry. *Journal of hepatology* **53**, 732-737 (2010).
66. Taylor, H.L., Buskirk, E. & Henschel, A. Maximal oxygen intake as an objective measure of cardio-respiratory performance. *Journal of applied physiology* **8**, 73-80 (1955).
67. Johnston, B.C.*, et al.* Comparison of weight loss among named diet programs in overweight and obese adults: a meta-analysis. *Jama* **312**, 923-933 (2014).
68. Hoddy, K.K.*, et al.* Meal timing during alternate day fasting: Impact on body weight and cardiovascular disease risk in obese adults. *Obesity* (2014).
69. Logue, J.*, et al.* Management of obesity: summary of SIGN guideline. *Bmj* **340**, c154 (2010).

70. Bakker, L.E., *et al.* A 5-day high-fat, high-calorie diet impairs insulin sensitivity in healthy, young South Asian men but not in Caucasian men. *Diabetes* **63**, 248-258 (2014).
71. Alligier, M., *et al.* Subcutaneous adipose tissue remodeling during the initial phase of weight gain induced by overfeeding in humans. *The Journal of clinical endocrinology and metabolism* **97**, E183-192 (2012).
72. Alligier, M., *et al.* Visceral fat accumulation during lipid overfeeding is related to subcutaneous adipose tissue characteristics in healthy men. *The Journal of clinical endocrinology and metabolism* **98**, 802-810 (2013).
73. Votruba, S.B. & Jensen, M.D. Insulin sensitivity and regional fat gain in response to overfeeding. *Obesity* **19**, 269-275 (2011).
74. Leibel, R.L., Rosenbaum, M. & Hirsch, J. Changes in energy expenditure resulting from altered body weight. *The New England journal of medicine* **332**, 621-628 (1995).
75. Schmidt, S.L., Kealey, E.H., Horton, T.J., VonKaenel, S. & Bessesen, D.H. The effects of short-term overfeeding on energy expenditure and nutrient oxidation in obesity-prone and obesity-resistant individuals. *International journal of obesity* **37**, 1192-1197 (2013).
76. Iggman, D., *et al.* Role of Dietary Fats in Modulating Cardiometabolic Risk During Moderate Weight Gain: A Randomized Double-Blind Overfeeding Trial (LIPOGAIN Study). *Journal of the American Heart Association* **3**(2014).
77. Johannsen, D.L., *et al.* Effect of 8 weeks of overfeeding on ectopic fat deposition and insulin sensitivity: testing the "adipose tissue expandability" hypothesis. *Diabetes care* **37**, 2789-2797 (2014).
78. Noakes, M., Foster, P.R., Keogh, J.B. & Clifton, P.M. Meal replacements are as effective as structured weight-loss diets for treating obesity in adults with features of metabolic syndrome. *The Journal of nutrition* **134**, 1894-1899 (2004).
