# Supplementary material for: Weight gain leads to greater adverse metabolic responses in South Asian compared with white European men: the GlasVEGAS study
Source: Nat Metab. 2024 Aug 16;6(8):1632–45. doi: 10.1038/s42255-024-01101-z (PMC11349579; doi:10.1038/s42255-024-01101-z)
Supplement: Supplementary file 2 — Reporting Summary [file 42255_2024_1101_MOESM2_ESM.pdf]

nature portfolio

Corresponding author(s): Jason Gill
Last updated by author(s): Jul 22, 2024

Reporting Summary

Nature Portfolio wishes to improve the reproducibility of the work that we publish. This form provides structure for consistency and transparency in reporting. For further information on Nature Portfolio policies, see our Editorial Policies and the Editorial Policy Checklist.

Statistics

For all statistical analyses, confirm that the following items are present in the figure legend, table legend, main text, or Methods section.

n/a	Confirmed	
☐	☒	The exact sample size (*n*) for each experimental group/condition, given as a discrete number and unit of measurement
☐	☒	A statement on whether measurements were taken from distinct samples or whether the same sample was measured repeatedly
☐	☒	The statistical test(s) used AND whether they are one- or two-sided<br>*Only common tests should be described solely by name; describe more complex techniques in the Methods section.*
☐	☒	A description of all covariates tested
☐	☒	A description of any assumptions or corrections, such as tests of normality and adjustment for multiple comparisons
☐	☒	A full description of the statistical parameters including central tendency (e.g. means) or other basic estimates (e.g. regression coefficient) AND variation (e.g. standard deviation) or associated estimates of uncertainty (e.g. confidence intervals)
☐	☒	For null hypothesis testing, the test statistic (e.g. *F*, *t*, *r*) with confidence intervals, effect sizes, degrees of freedom and *P* value noted<br>*Give P values as exact values whenever suitable.*
☒	☐	For Bayesian analysis, information on the choice of priors and Markov chain Monte Carlo settings
☒	☐	For hierarchical and complex designs, identification of the appropriate level for tests and full reporting of outcomes
☐	☒	Estimates of effect sizes (e.g. Cohen's *d*, Pearson's *r*), indicating how they were calculated

Our web collection on statistics for biologists contains articles on many of the points above.

Software and code

Policy information about availability of computer code

Data collection	jMRUI (for magnetic resonance spectroscopy); ImageJ (for adipocyte sizing)
Data analysis	Statistica (version 10.0, StatSoft Inc, Tulsa, OK) and Minitab (version 20.0, Minitab, State College, PA, USA)

For manuscripts utilizing custom algorithms or software that are central to the research but not yet described in published literature, software must be made available to editors and reviewers. We strongly encourage code deposition in a community repository (e.g. GitHub). See the Nature Portfolio guidelines for submitting code & software for further information.

Data

Policy information about availability of data

All manuscripts must include a data availability statement. This statement should provide the following information, where applicable:

- Accession codes, unique identifiers, or web links for publicly available datasets
- A description of any restrictions on data availability
- For clinical datasets or third party data, please ensure that the statement adheres to our policy

Anonymised data generated during and analysed during the current study will be available on request. The full dataset contains several indirect identifiers so unfortunately cannot be shared publicly (beyond the aggregate data presented in the paper) in line with current guidance (see DOI: 10.1186/1745-6215-11-9).

Research involving human participants, their data, or biological material

Policy information about studies with human participants or human data. See also policy information about sex, gender (identity/presentation), and sexual orientation and race, ethnicity and racism.

Reporting on sex and gender	The data in this study was from men only and participants are referred to as men in the manuscript.
Reporting on race, ethnicity, or other socially relevant groupings	The study includes two distinct ethnic groups: White Europeans - defined as self-report of both parents of white European origin) and South Asian - defined as self-report of both parents of Indian, Pakistani, Bangladeshi or Sri Lankan origin.
Population characteristics	Participants were men, aged 18-45 years, with BMI <25 kg.m2
Recruitment	Participants were recruited via personal contacts and local advertising. As this was an intervention trial where the main outcome was ethnic differences in within-participant changes from from baseline with weight gain, rather than to establish whether differences in baseline characteristics cross-sectionally, and participants were of similar age and BMI at baseline and all lived in the same UK city, we don't consider that the specific method of participant recruitment would be a major source of bias in this study.
Ethics oversight	University of Glasgow College of Medical, Veterinary and Life Sciences Ethics Committee

Note that full information on the approval of the study protocol must also be provided in the manuscript.

Field-specific reporting

Please select the one below that is the best fit for your research. If you are not sure, read the appropriate sections before making your selection.

☒ Life sciences ☐ Behavioural & social sciences ☐ Ecological, evolutionary & environmental sciences

For a reference copy of the document with all sections, see nature.com/documents/nr-reporting-summary-flat.pdf

Life sciences study design

All studies must disclose on these points even when the disclosure is negative.

Sample size	The sample size was based on power to detect a 1-SD difference for change in outcomes of interest with weight-gain between groups, based on the typically ≥ 1-SD difference in postprandial insulin response, fat mass and adipocyte size observed in previous comparisons between normoglycaemic South Asian and White European adults 4,12. Twenty-three participants per group provides 90% power to detect this magnitude of difference, with 80% power provided with 17 per group. To achieve 23 completers per group allowing for drop-out, we aimed to recruit 30 participants per group (i.e. 30% over-recruitment). Our final achieved power to detect a 1-SD difference with 21 white European and 14 South Asian participants completing the study was 80.3%.
Data exclusions	There were no data exclusions. All available data points were included in the analysis.
Replication	This was a human experimental study where each individual was measured at baseline and following the intervention. Each participant completed the intervention once. Given the nature of the study (weight gain over several weeks, followed by weight loss to baseline weight over several weeks post-intervention) it would not be ethical to ask participants to undergo the intervention more than once.
Randomization	Not applicable - participants all undertook the same intervention.
Blinding	There was no group allocation as all participants underwent the same intervention. Biochemical analyses, and adipocyte sizing and gene expression analyses were blinded. Data collection and statistical analysis were not performed blind to the conditions of the experiments.

Reporting for specific materials, systems and methods

We require information from authors about some types of materials, experimental systems and methods used in many studies. Here, indicate whether each material, system or method listed is relevant to your study. If you are not sure if a list item applies to your research, read the appropriate section before selecting a response.

Materials & experimental systems

n/a	Involved in the study	
☒	☐	Antibodies
☒	☐	Eukaryotic cell lines
☒	☐	Palaeontology and archaeology
☒	☐	Animals and other organisms
☐	☒	Clinical data
☒	☐	Dual use research of concern
☒	☐	Plants

Methods

n/a	Involved in the study	
☒	☐	ChIP-seq
☒	☐	Flow cytometry
☒	☐	MRI-based neuroimaging

Clinical data

Policy information about clinical studies

All manuscripts should comply with the ICMJE guidelines for publication of clinical research and a completed CONSORT checklist must be included with all submissions.

Clinical trial registration

ClinicalTrials.gov (NCT02399423)

Study protocol

Ethics application including study protocol is available in supplementary materials.

Data collection

Participants in the study lived in the vicinity of Glasgow, United Kingdom and data collection was undertaken in the research laboratories in the College of Medical, Veterinary and Life Sciences at the University of Glasgow, Glasgow, United Kingdom. Participant recruitment occurred between 2015 and 2017.

Outcomes

This was a mechanistic study aimed at uncovering ethnic differences in the extent of metabolic and physiological changes in response to weight gain between South Asians and Europeans to uncover potential mechanisms underpinning the elevated risk of type 2 diabetes in the former group. To this end, we specified a broad range of primary outcomes to cover the major potential physiological and metabolic changes in response to weight gain that could potentially differ between groups.

The aims of this basic human experimental study were therefore to compare the effects of induced weight-gain in SA and WE men without overweight or obesity at baseline on: i) body composition and subcutaneous, visceral and ectopic fat accumulation; ii) metabolic responses to a physiologically-relevant mixed-meal challenge; and iii) adipocyte morphology. We also sought to investigate factors contributing to ethnic differences in weight-gain induced changes in indices of insulin sensitivity/insulin resistance and estimated insulin secretion/[illegible]-cell function, and undertook an exploratory analysis of ethnic differences in expression of adipocyte genes related to lipid metabolism, differentiation, tissue stress response and inflammation.

Differences between ethnic groups at baseline were analysed using unpaired t-tests. The effects of weight-gain (main effect of weight-gain) and differences between ethnic groups in changes due to weight-gain (weight-gain x ethnicity interaction) were analysed by two-way ANOVA (ethnicity x time), with repeated-measures on the time factor (baseline, post-intervention). To assess whether ethnic differences in responses to weight gain were independent of any ethnic differences in baseline values, two-way ANCOVAs were performed, with baseline values included as a co-variate in the models. To help understand factors contributing to the ethnic differences in the changes in fasting insulin concentrations, the postprandial insulin response, HOMAIR, the Matsuda Insulin Sensitivity Index, insulin AUC/glucose AUC, and ISSI-2 with weight gain (change in glucose and triglyceride responses with weight-gain did not differ between ethnic groups), uni- and multi-variable linear regressions between the change in these variables with weight gain, with body composition variables, fitness and physical activity variables, adipocyte size variables were explored. Uni-variable associations between adipocyte gene expression and adipocyte size variables were also investigated in exploratory analyses. All tests were two-sided and statistical significance was accepted at a nominal value of $p<0.05$. In this basic human experimental study, our primary aims were around ethnic differences in responses to weight gain in three 'families' of data – anthropometric/body composition (10 outcomes, excluding change in weight and BMI, which the same in both groups, by design), metabolic (12 outcomes), and adipocyte morphology (10 outcomes). We also reported data on changes in cardiorespiratory fitness and physical activity (8 outcomes) and undertook an exploratory analysis of ethnic differences in expression of adipocyte genes related to lipid metabolism, adipocyte differentiation, tissue stress response and inflammation (25 outcomes). To reduce the familywise risk of type 1 statistical error, we therefore used the method described by Tukey et al (Bonferroni adjustment is considered too conservative when there are multiple high-correlated outcomes as is the case in the present study), to adjust the p-value for statistical significance from 0.05 to 0.02 for body composition, metabolic, adipocyte morphology and fitness/physical activity outcomes (assuming 12 outcomes in each family) and to 0.01 for adipocyte gene expression outcomes (25 outcomes).

Plants

Seed stocks	N/A
Novel plant genotypes	N/A
Authentication	N/A
